# Revisiting the Soybean GmNAC Superfamily

**DOI:** 10.3389/fpls.2018.01864

**Published:** 2018-12-18

**Authors:** Bruno P. Melo, Otto T. Fraga, José Cleydson F. Silva, Dalton O. Ferreira, Otávio J. B. Brustolini, Paola A. Carpinetti, Joao Paulo B. Machado, Pedro A. B. Reis, Elizabeth P. B. Fontes

**Affiliations:** ^1^National Institute of Science and Technology in Plant-Pest Interactions, Bioagro, Universidade Federal de Viçosa, Viçosa, Brazil; ^2^Departamento de Bioquímica e Biologia Molecular/BIOAGRO, Universidade Federal de Viçosa, Viçosa, Brazil; ^3^Agronomy Institute, Universidade Federal de Viçosa, Florestal, Brazil

**Keywords:** NAC, soybean, phylogenetic analysis, senescence-associated genes, genome-wide expression profiling, GmNAC superfamily, transcriptional factors

## Abstract

The *NAC* (NAM, ATAF, and CUC) genes encode transcription factors involved with the control of plant morph-physiology and stress responses. The release of the last soybean *(Glycine max)* genome assembly (Wm82.a2.v1) raised the possibility that new NAC genes would be present in the soybean genome. Here, we interrogated the last version of the soybean genome against a conserved NAC domain structure. Our analysis identified 32 putative novel NAC genes, updating the superfamily to 180 gene members. We also organized the genes in 15 phylogenetic subfamilies, which showed a perfect correlation among sequence conservation, expression profile, and function of orthologous *Arabidopsis thaliana* genes and NAC soybean genes. To validate our *in silico* analyses, we monitored the stress-mediated gene expression profiles of eight new NAC-genes by qRT-PCR and monitored the GmNAC senescence-associated genes by RNA-seq. Among ER stress, osmotic stress and salicylic acid treatment, all the novel tested GmNAC genes responded to at least one type of stress, displaying a complex expression profile under different kinetics and extension of the response. Furthermore, we showed that 40% of the GmNACs were differentially regulated by natural leaf senescence, including eight (8) newly identified GmNACs. The developmental and stress-responsive expression profiles of the novel NAC genes fitted perfectly with their phylogenetic subfamily. Finally, we examined two uncharacterized senescence-associated proteins, GmNAC065 and GmNAC085, and a novel, previously unidentified, NAC protein, GmNAC177, and showed that they are nuclear localized, and except for GmNAC065, they display transactivation activity in yeast. Consistent with a role in leaf senescence, transient expression of GmNAC065 and GmNAC085 induces the appearance of hallmarks of leaf senescence, including chlorophyll loss, leaf yellowing, lipid peroxidation and accumulation of H_2_O_2_. GmNAC177 was clustered to an uncharacterized subfamily but in close proximity to the TIP subfamily. Accordingly, it was rapidly induced by ER stress and by salicylic acid under late kinetic response and promoted cell death *in planta*. Collectively, our data further substantiated the notion that the GmNAC genes display functional and expression profiles consistent with their phylogenetic relatedness and established a complete framework of the soybean NAC superfamily as a foundation for future analyses.

## Introduction

As sessile organisms, plants are exposed to various environmental adverse conditions including drought, salinity, pest, and pathogen attacks. To cope with these stress conditions, plants have evolved a sophisticated mechanism for the perception of stimuli and transduction of signals, which leads to the reprogramming of gene expression for adaptation. The knowledge about the molecular mechanisms by which plants avoid the stresses conditions and adapt themselves to maintain yield may allow the identification of molecular targets for genetic engineering of stress tolerance. In these cascades of signals, the transcription factors (TF) are among the most promising molecular targets to promote adaptive plant physiology changes.

Among the TF families, the plant-specific NAC (an acronym for NAC, ATAF and CUC) superfamily comprises one of the largest family of TFs (Shao et al., 2015). NAC TFs display a conserved amino-terminus, encompassing the DNA binding domain, and a rather variable carboxyl-terminus, which harbors a transcriptional regulatory domain with either repressing or activating functions (Aida et al., 1997; Olsen et al., 2005; Nakashima et al., 2011). A subset of NAC proteins may also exhibit protein binding activity and an additional transmembrane domain present in the membrane-tethered NAC proteins (Tran et al., 2009; Seo et al., 2010). The DNA binding domain, the NAC domain, which has been used as a template for the identification of NAC genes in different plant species, contains 150–200 conserved amino acids split into two exons that are further divided into five conserved subdomains, designated A-E.

The NAC genes were first identified as major players in plant development. Several NAC members have been functionally characterized in floral development (Sablowski and Meyerowitz, 1998), apical meristem formation (Hegedus et al., 2003; Hibara et al., 2003), lateral root development (Xie et al., 2000; Hao et al., 2011; Quach et al., 2014), growth hormone signaling (Xie et al., 2000; Fujita et al., 2004), cell-cycle control (Kim et al., 2006), secondary cell wall thickening and biogenesis (Mitsuda et al., 2005, 2007; Zhong et al., 2006; Mitsuda and Ohme-Takagi, 2008; Dong et al., 2013), and senescence (Guo and Gan, 2006; Kim et al., 2016). High-resolution temporal expression profiles revealed that a large fraction of NAC TFs is differentially expressed during several stages of natural leaf senescence in Arabidopsis (Breeze et al., 2011), suggesting that they play a crucial role in the regulation of senescence. Although functional information in developmental programmed leaf senescence is available for family members of different plant species (Guo and Gan, 2006; Kim et al., 2009; Balazadeh et al., 2010, 2011; Yang et al., 2011; Lee et al., 2012; Wu et al., 2012; Li et al., 2016b; Pimenta et al., 2016), demonstrating the biotechnological potential of the senescence NAC genes for seed yield (Liang et al., 2014), the repertoire of differential expressed senescence-associated NAC genes in the genome of relevant crops, including soybean, maize, and rice, remains to be determined.

From the pioneering studies of NAC TFs in developmental programs, the NAC TF function has expanded to include key regulators of plant defenses against environmental stresses; thereby, emerging as potential targets for engineering stress tolerance in crops (Nakashima et al., 2011; Puranik et al., 2012; Shao et al., 2015). The NAC TFs have also been shown to play relevant roles in the control of plant defenses against pathogens (Puranik et al., 2012), programmed cell death (Faria et al., 2011; Mendes et al., 2013; Mao et al., 2018) and endoplasmic reticulum stress (Yang et al., 2014a,b; Reis et al., 2016).

Concerning abiotic stress responses, the NAC genes have been primarily studied for drought tolerance. The functions of NAC TFs in the improvement of drought tolerance was firstly demonstrated in Arabidopsis by the overexpression of the *ANAC019, ANAC055*, and *ANAC072* genes (Tran et al., 2004, 2007). Abiotic stress-related functions of NAC TFs in various plant species, including important crops such as rice and wheat, have also been reported (Nakashima et al., 2007, 2011; Puranik et al., 2012), even in field trials (Hu et al., 2006; Redillas et al., 2012). Likewise, in soybean, GmNAC020 has been demonstrated to promote abiotic stress tolerance and lateral root formation in transgenic plants (Hao et al., 2011). Furthermore, by examining contrasting soybean genotypes for drought tolerance, a positive correlation between the expression of a subset of GmNACs and drought tolerance has been reported (Kim et al., 2006), further supporting the notion that GmNACs may be selected as a target for improving drought tolerance. The drought-sensitive (B217 and H228) and the drought-tolerant (Jindou 74 and 78) soybean cultivars have also been used to select GmNACs highly expressed in the drought-resistant soybean varieties (Hussain et al., 2017). The potential of NAC genes for tolerance to high salinity and cold has also been investigated in several plant species (Hu et al., 2006, 2008; Nakashima et al., 2007; Zheng et al., 2009; Takasaki et al., 2010; Hao et al., 2011; Song et al., 2011; Cao et al., 2017). However, most NAC transcription factors have not yet been functionally characterized, and the extension and complexity of the NAC family in the plant kingdom have not been thoroughly examined.

In soybean, the first study of NAC genes included the molecular cloning of six NAC genes designated as GmNAC1-6 (Meng et al., 2007). Subsequently, the expression of these genes in response to various stress conditions and hormone treatments was analyzed and the identification of 111 NAC genes in the soybean genome was reported (Pinheiro et al., 2009). These studies were expanded to cover the expression of 31 GmNAC genes at the seedling stage and under different stress conditions (Tran et al., 2009). A more complete genome-wide survey identified 152 full-length GmNAC TFs, including 11 membrane-bound members, and 31 drought-responsive GmNACs with some degree of tissue-specificity (Le et al., 2011). However, the dynamic of drought-responsiveness of GmNACs was found to be complex and integrated with tissue-specific and/or developmental stage-dependent expression profiles of these genes (Le et al., 2012). More recently, the number of membrane-bound NAC transcription factor (NTL) genes was expanded from 11 to 15 genes, from which seven duplicated genes were identified (Le et al., 2011; Li et al., 2016b). An evolutionary and functional analysis of the soybean membrane-bound NAC transcription factor genes indicated that their membrane release is essential for function and the duplicate genes diverge functionally, which contributes as an evolutionary driving force for the retention of these GmNTL duplicate genes (Li et al., 2016b). These recent studies together with the release of the last version of the soybean genome (version V11) indicate that the complete repertoire of the NAC genes in the soybean genome remains to be described.

The size of the NAC family has also been examined in Arabidopsis (111 genes), rice (151), maize (152), and other plant species (Shao et al., 2015). The family has been divided into subgroups based on phylogenetic relatedness, showing a clear relationship between structure and expression for representatives of each subgroup (Pinheiro et al., 2009). A common theme that has emerged from these genome-wide analyses and expression-profiling studies is that the plant NAC family is structurally and functionally conserved with a high degree of ascertaining for functional predictions of putative orthologs from different species. Accordingly, the full determination of the NAC superfamily among plant species is expected to accelerate the functional characterization of this extensive family of plant-specific TFs in relevant crops to which molecular tools are limited and hence functional studies are more difficult to pursue. In this study, we revisited the GmNAC family by using the most recent assembly of the soybean genome, which allowed us to perform a phylogenetic reconstruction of the family. Through this analysis, we identified 32 additional members of the soybean NAC family, which were clusters in the previously characterized subgroups of GmNACs based on phylogeny and expression analysis. We also confirmed the size of the membrane-tethered GmNACs, identified the GmNAC-associated senescence genes by genome-wide expression analyses and performed functional assays of cell death-associated gene representatives.

## Materials and Methods

### Phylogenetic Reconstruction of the Soybean NAC Superfamily

A revised soybean genome assembly (version Wm82.a2.v1-v11.0) released in 2015 in the Pthytozome database (www.phytozome.jgi.doe.gov) led us to investigate the existence of new NAC genes (complete ORFs) in the soybean genome. We used the Pfam (29.0-http://pfam.xfam.org/) NAC domain as the prototype sequence for searching against all deduced protein sequences from the last soybean genome assembly Wm82.a2.v1-v11.0 in the FASTA format. The sequences comparison was performed in InterProScan 5 (http://www.ebi.ac.uk/Tools/pfa/iprscan5/) software, and newly identified proteins, not described by Le et al. (2011), were grouped by ID number (Glyma v11.0 pattern), size (amino acid number), NAC domain position (the first and the least amino acids that characterize NAC domain), *E*-value (statistical significance), and transmembrane-domain probability (Supplementary Table 1). Only the sequences that contained a full-length NAC domain were used for the multiple alignments and phylogenetic analysis.

For the phylogenetic reconstruction of the soybean NAC superfamily, the *Arabidopsis thaliana* deduced NAC protein (available in Phytozome) sequences were used in a global alignment with all soybean full-length NAC sequences. The alignment was conducted using Muscle algorithm (http://www.ebi.ac.uk/Tools/msa/muscle/) and allowed not only a sequence-based comparison but also a function-based comparison, as *A. thaliana* NAC transcription factors have been well-characterized. To refine the phylogenetic reconstruction, *A. thaliana* ortholog proteins from EggNOG database (http://eggnogdb.embl.de/#/app/home) were individually compared with soybean proteins. The sequences were used to reconstruct the NAC soybean and Arabidopsis phylogenetic tree by the maximum likelihood statistical method with 10.000 bootstraps. The tree was edited using the FigTree (http://tree.bio.ed.ac.uk/software/figtree/) software.

### Distribution of the New NAC Genes in the Soybean Chromosomes

To determine the distribution of the NAC genes along 20 soybean chromosomes, the NAC domain sequence available on InterProScan database (code: IPR003441) was used as a guide and the analysis was performed using PLAZA 3.0 (Plant Comparative Genome-http://bioinformatics.psb.ugent.be/plaza/versions/plaza_v3_dicots/genome_mapping/index), which provides a schematic diagram of soybean chromosomes and the position of the guide-related genes. PLAZA's diagram and Phytozome ID NAC numbers were used to organize the superfamily in a schematic chromosome map.

### Synteny Analysis

The duplicated GmNAC CDS sequences were aligned with Clustal Omega (https://www.ebi.ac.uk/Tools/msa/clustalo) to predict the duplications/ paralogous pairs. The R package seqinr (http://seqinr.r-forge.r-project.org) was used to calculate the non-synonymous (Ka) and synonymous (Ks) rate ratio of the paralog genes. We used the same formula applied by Zhang et al. (2018) to calculate the divergence time [*T* = Ks/(2 × 6.1 × 10^−9^) × 10^−6^ Mya].

### Plant Growth Conditions and Stress Treatments

Soybean (*Glycine max*-Conquista) seeds were germinated and grown under greenhouse conditions (12 h of light, 15–30°C, 70% relative humidity). At the V2/V3 developmental stage, the roots were immersed in Hoagland Hydroponic Solution supplemented with 10% (w/v) PEG (molecular weight 8,000), 5 μg/mL tunicamycin (TUN) or 5 mM salicylic acid (SA) to induce osmotic, endoplasmic reticulum and biotic stress conditions, respectively. Dimethyl sulfoxide (DMSO) was used as a control for TUN treatment. Leaf disks from stressed and control leaves were collected at 0.5, 2, and 12 h post-treatment (for PEG treatment, a 24 h harvest time was included), immediately frozen in liquid nitrogen, and stored at −80°C until processing. For the analysis of gene expression of putative genes in different soybean vegetative tissues, samples of leaf disks (1 cm diameter), pivotal and lateral roots, stem's segments (1 cm), entire flowers and pods segments were collected and frozen in liquid nitrogen from R2/R3 developmental stage plants.

### RNA Extraction and cDNA Synthesis

For gene expression profile of putative new NAC genes under stress conditions, total RNA was extracted from the leaf disks using Trizol (ThermoFisher). For expression analysis, we used four biological replicates of a pool of 3 plants for each stress treatment. cDNA synthesis was performed with 4 μg of total RNA, 10 μM oligoDT (18T), 10 mM dNTPs, and 200U of MMLV reverse transcriptase, according to the manufacturer's instructions (ThermoFischer). The expression profile of NAC genes was also determined in different soybean tissues. For this analysis, three biological replicates of a pool of 5 plants for each vegetative collected tissue were used and 2 μg of RNA were used for cDNA synthesis. For the analysis of differential expression during senescence, cDNAs were prepared from leaves of plants 20 days after germination (DAG) and 80 DAG. In this case, 3 biological samples and 3 technical replicates for each treatment were used. cDNAs from RNA of stressed leaf disks were used for the isolation of ORFs from the drought-repressed GmNAC065, drought-induced GmNAC085 (Carvalho et al., 2014a) genes and the newly identified GmNAC177 (this work).

### Construction of Plasmids

All recombinant plasmids were obtained through the GATEWAY system (ThermoFisher). GmNAC065, GmNAC085, GmNAC177 coding regions were amplified by PCR from cDNA of stressed leaves using gene-specific primers harboring appropriate extensions and introduced by recombination into the entry vectors pDONR201 and pDONR207 and then transferred to pDEST22, pDEST32, and pEARLEY103/104. The PCR amplicons were purified from ethidium bromide-staining agarose gel by QIAquick Gel extraction kit (QIAGEN). After spectrophotometric quantification, 50 fmol of empty vector and PCR product were incubated at 25°C—with 1 μL of BpClonase (ThermoFisher) for 8 h. The same conditions were used to transfer the insert from pDONR201 or pDONR2007 to pDEST22, pDEST32 or pEARLEY103/104 using LrClonase enzyme (ThermoScientific). Recombination plasmids were transformed into *E. coli* DH5α and selected on LB-agar medium supplemented with 10 μM gentamicin (for pDONR207 or pDEST32), 100 μM kanamycin (for pDONR201 or pEARLEY103/104) or 100 μM ampicillin (for pDEST22). The resulting clones were confirmed by sequencing. Primers used and generated clones are described in Supplementary Tables 2, 3, respectively.

### Transient Expression and Subcellular Localization of Fused Proteins in *Nicotiana benthamiana*

To confirm the nuclear subcellular localization of NAC065, NAC085, and NAC177, the DNA constructions 35S:NAC065-GFP, 35S:NAC117-GFP or 35S:YFP-NAC085 were used to transfect *Nicothiana benthamiana* leaves by agroinfiltration. *Agrobacterium tumefaciens* GV3101 strain was transformed with pUFV2830, pUFV3007, or pUFV3008 (Supplementary Table 3) the bacterial suspension at O.D_600_ = 0.5 was mechanically inoculated in leaf abaxial surface. All three constructions were co-infiltrated with AtWWP1-mCherry (pUFV 2224), an *A. thaliana* nuclear marker gene (Silva et al., 2015; Calil et al., 2018). Three days after inoculation, 1 cm^2^ infiltrated leaf sections were analyzed under Zeiss LSM510 META Laser Confocal Microscopy. YFP and GFP were excited by argon/helium-neon laser system in 488 nm wavelength, and emission was collected in 500–530 nm band-pass filter, while mCherry was excited in 543 nm wavelength and emission collected in 596–638 nm band-pass filter. Images were captured and treated in LSM Image Browser 4 (Carl-Zeiss) software.

### Transactivation Assay in Yeast and Yeast-Two-Hybrid Assay

For transactivation assay in yeast cells, the NAC coding regions were fused to the GAL4 binding domain and transactivation of reporter genes were assayed in *Saccharomyces cerevisiae*, strain AH109 (MATa, Trp1-901, leu2-3, 112, ura3-52, his3-200, gal4Δ, LYS2::GAL1UAS-GAL1TATA-HIS3, MEL1 GAL2UAS-GALTATA::MELUAS- MEL1TATA-lacZ). AH109 competent cells were transformed with pBD-NAC065, pBD- NAC085, and pBD-NAC117 (Supplementary Table 3) or pBD (control), along with 100 μg of salmon sperm carrier DNA (ssDNA), using the lithium acetate/polyethylene glycol (PEG) method. Transactivation activity was monitored by placing different optical dilutions (O.D_600_ = 1.0, 0.1, and 0.01) of the transformant suspension on synthetic dropped (SD) medium lacking leucine and histidine but supplemented with 10 mM 3-amino-1,2,4-triazole (AT) *HIS3* gene-product competitive inhibitor and cultured for 3 days at 28°C. For the yeast two-hybrid assay, AH109 was cotransformed with pAD-NAC fusions and pBD-NAC fusions (Supplementary Table 3) and interactions were monitored by the ability of the reporter strain to grow on SD/-leu/-tryp/-his selective medium supplemented with 3-amino-1,2,4-triazole (10 mM).

### Quantitative RT-PCR

Real-time RT-PCR assays were performed with an ABI 7500 instrument (ThermoFisher) using SYBR Green PCR Master Mix (Life Technologies), gene-specific primers (Supplementary Table 4) and cDNA from stressed seedlings and R2/R3 plants under normal growth conditions. Three biological replicates (three unstressed or stressed seedlings) were used to obtain two independent mRNAs pools for the quantitative RT-PCR data with two technical replicates. Relative gene expression was quantified using the comparative 2^−ΔΔCt^ or ^2−ΔCt^ method for the analysis of stressed and control plants, respectively. *UKN-2* was chosen as the normalizer, endogenous control gene (Libault et al., 2008). Analysis of expression of *SMP* (seed maturation protein), a drought-induced gene, *CNX* (calnexin) and *PDI* (protein disulfide isomerase*)*, ER stress-responsive genes, and *PR-4 (*pathogenesis-related 4*)*, an SA-induced gene, were used as control genes for the respective stress treatments. The amplification reactions were performed as follows: 2 min at 50°C, 10 min at 95°C, and 40 cycles of 94°C for 15 s and 60°C for 1 min. The relative gene expression quantification was converted into a heat map using MORPHEUS (https://software.broadinstitute.org/morpheus/) software. Ct data and standard deviation were organized in Supplementary Tables 5, 6).

### RNA Sequencing Experiment

Fresh tissue (six 0.9 cm diameter disks) from the middle leaflet of third trifoliate and seventh trifoliate leaf of soybean plants was collected and stored at −80°C. Leaves were collected in two plant stages, vegetative stage three (V3) and reproductive stage seven (R7). For RNA-sequencing, wild-type plants (BR16) were used in biological triplicates. Total RNA was extracted using the Trizol reagent (ThermoFischer), as recommended by the manufacturer, followed by precipitation with isopropanol. The integrity and quality of the extracted RNA were monitored using the Agilent RNA 6000 assay on the Agilent Bioanalyzer 2100 system. The quantification of total RNA was obtained by Quant-iTRiboGreen RNA Assay Kit (ThermoFisher). The sequencing libraries were prepared with TruSeq® Stranded Total RNA Sample Preparation Kit (Illumina) using the LowSample (LS) Protocol, according to the manufacturer's recommendations. Additionally, ribosomal RNA was depleted through Ribo-Zero Deplete beads. One microgram total RNA per sample was used as input material for the RNA sample preparation, after treatment with DNase I Amplification Grade (ThermoFisher). The products were purified using AMPure XP system (Beckman Coulter, Beverly, MA) and quantified using the Agilent High Sensitivity DNA assay on the Agilent Bioanalyzer 2100 system.

The clustering of the index-coded samples was performed using the cBot Cluster Generation System with TruSeq PE Cluster Kit v3-cBot for 101 cycle paired-end reads (Illumina), according to the manufacturer's recommendations. The samples were sequenced on Illumina Hiseq 2500 platform (Illumina), and 100 bp paired-end reads were generated. The run was performed using the High Output mode.

For differential gene expression (DGE) analysis, it was applied the R/Bioconductor package DESeq2 for the normalization and statistical test (Love et al., 2014). The *p*-value was corrected by False Discovery Rate (FDR) and significance was considered with *p* < 0.05. To minimize false positive DE genes, the output of DESeq2 was applied to the Bioconductor package IHW that was used to do an independent hypothesis weighting (IHW). For the downstream analysis, the data were converted to SQL tables stored in PostgreSQL version 10.1 database. The Gene Ontology (GO) analysis was performed by the R/Bioconductor package GOstats (Falcon and Gentleman, 2007). For gene enrichment of GO terms, we used hypergeometric tests. The significant *p*-value for enrichment was *P* < 0.01. Pathway analyses were performed by the R/Bioconductor package path view (Luo and Brouwer, 2013). The orthology table was used by comparing *Glycine max* to the matched gene at *A. thaliana* database. Hence, the maps referred to Arabidopsis organism was applied using KEGG graphics. RNA-sequencing data have been deposited in the Gene Expression Omnibus under accession number GSE122915 (https://www.ncbi.nlm.nih.gov/geo/query/acc.cgi?acc=GSE122915).

### Transient Expression of GmNAC065, GmNAC085, and GmNAC177 in *Nicotiana benthamiana* and Cell Death Progression Analyses

Agrobacterium-mediated transient expression of NAC fusion in *N. Benthamiana* leaf epidermal cells was performed as described (Silva et al., 2015). GmNAC081 and NRP-B were used as positive control for the induction of cell death (Costa et al., 2008; Faria et al., 2011). Three days after infiltration, leaf disks were collected and used to quantify total chlorophyll and TBA-reactive compounds and for western-blotting of NAC proteins from total protein extracts. The leaves were used to observe the development of typical symptoms of cell death and to monitor hydrogen peroxide production by DAB staining.

Total chlorophyll content was determined spectrophotometrically after quantitative extraction from leaves (Wellburn, 1994). Briefly, leaf disks were weighted, and total chlorophyll was extracted with absolute ethanol, transferred to a dark tube, centrifuged at 12,000 g and quantified from the supernatant by spectrophotometry. Absorption values were collected at 645 nm and 663 nm and total chlorophyll expressed as μg/g.

TBA-reactive compounds were quantified as described by Cakmak and Horst (1991). Approximately 200 mg of leaves were homogenized in 2 ml of 0.1% (v/v) trichloroacetic acid (TCA) and then centrifuged at 12,000 g for 15 min. All steps were performed at 4°C. A 500 μL-aliquot of the supernatant was added to 1.5 ml of 0.5% (v/v) thiobarbituric acid (TBA) in 20% (v/v) TCA, and the samples were incubated at 90°C for 20 min. The reaction was stopped by incubation on ice followed by centrifugation for 4 min. The absorbance of the supernatant was measured at 532 nm, and the concentration of TBA-reactive compounds was calculated using the molar absorption coefficient of 155 mM^−1^ cm^−1^, according to Heath and Packer (1968).

For hydrogen peroxide staining, the infiltrated leaves were incubated during 8 h in a 3,3-diaminobenzamidine (DAB) solution (1 mg/mL-pH 3.8) under continuous agitation. After staining, the leaves were immersed in a 100% hot ethanol bath, rehydrated at 80% ethanol bath, followed by 70% ethanol, 50% ethanol and, finally, water.

### Statistical Analyses

All statistical analyses (ANOVA and *T*-test) were performed using the online software ANOVA calculator and *T*-test calculator (http://www.socscistatistics.com).

## Results

### Phylogenetic Reconstruction of the GmNAC Superfamily: Identification of New Members in the Soybean Genome

**Figure 1:**
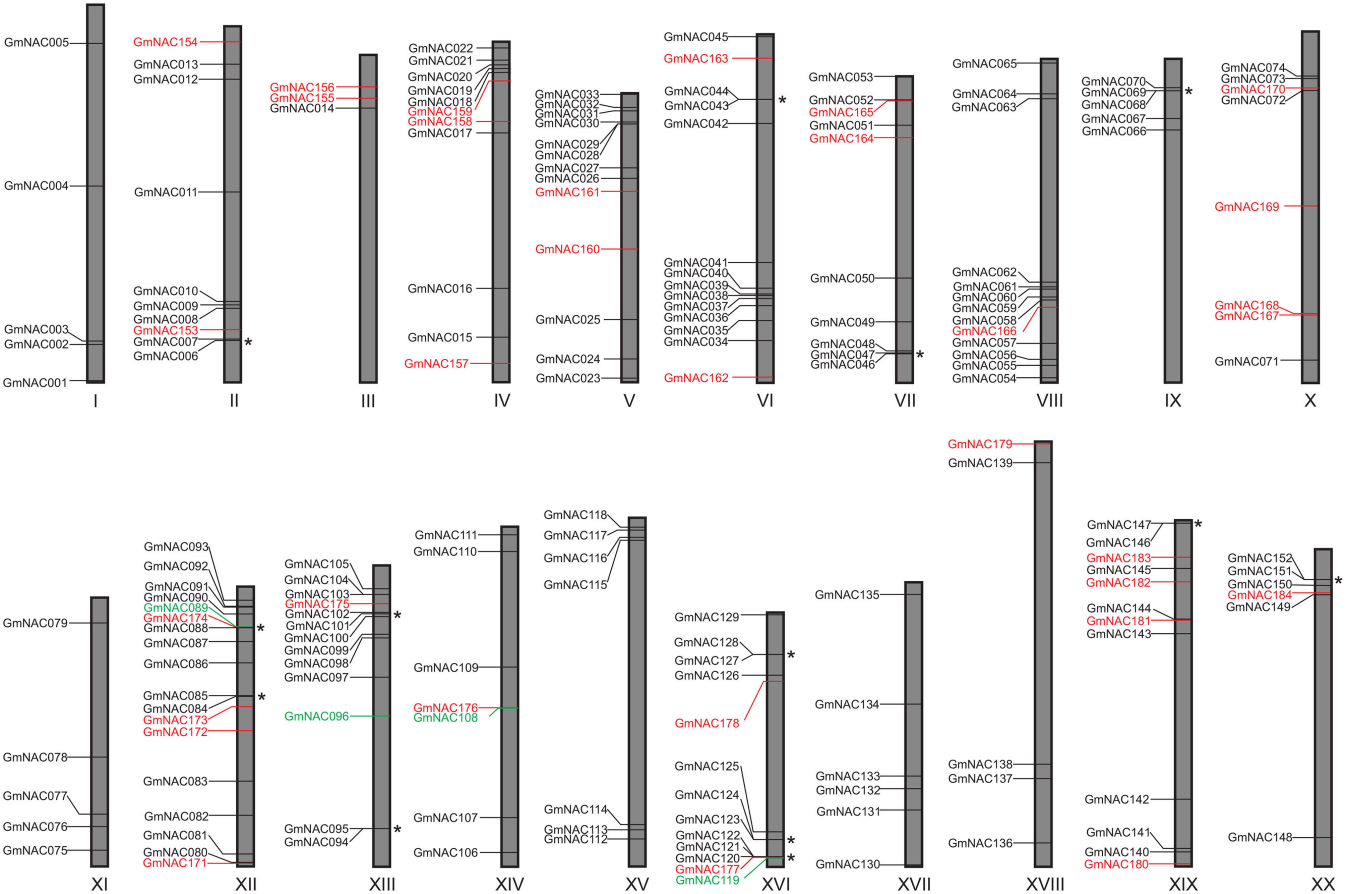
Graphical representation of the location for the GmNACs on the soybean chromosomes. Previously described genes are indicated in black, and the newly identified genes are in red. GmNACs in green represent the previously identified ones, which were no longer found in the soybean genome reassembly. The asterisks indicate tandem duplicated genes.

Due to the relevance of the NAC gene family in developmental programs and stress response, this large family of plant-specific TFs has been extensively studied in different plant species. The most complete description of the soybean superfamily was provided by Le et al. (2011), which described 152 members (with complete ORFs), from which 58 members were associated with dehydration response. Here, we used the NAC domain for searching against all deduced protein sequences from the last version of the soybean genome Wm82.a2.v1-v11.0. Our current wide-genome analysis revealed 32 new NAC candidate genes, not previously described (Le et al., 2011). A complete inventory of the GmNAC superfamily is presented in Supplementary Table 1, in which new, previously unidentified, members are highlighted in yellow. The nomenclature Glyma was provided by Phytozome and identifies the gene locus position among 20 soybean chromosomes. For standardization in scientific communication, in the previous nomenclature from Le et al. (2011), the NAC genes received an increasing number after the GmNAC prefix taking into consideration the order of the chromosome and locus position. To maintain the nomenclature of the 152 previously described NAC genes, the new NAC genes were numbered successively after GmNAC152 using the same system as in Le et al. (2011) (Table 1, Figure 1).

**Table 1:** New, previously unidentified, putative NAC genes.

**Glyma v11.0**	**Name**	**Length**	**NAC-domain**	***E*-value**	TM^***^	**Chromos**	**Subfamily**
Glyma.02G050100.1.p	GmNAC153	362	6–135	6.8E-53		II	VND-NAC
Glyma.02G284300.1.p	GmNAC154	320	22–149	1.0E-47		II	ONAC022
Glyma.03G164200.1.p	GmNAC155	296	10–138	6.7E-48		III	NAM
Glyma.03G179600.1.p	GmNAC156	287	11–138	9.4E-48		III	SNAC-B
Glyma.04G014900.1.p	GmNAC157	350	22–149	1.4E-49		IV	NAM
Glyma.04G175800.1.p^*^	GmNAC158	78	02–78	1.3E-21		IV	Senu5
Glyma.04G199000.1.p	GmNAC159	169	28–73	4.0E-11		IV	Senu5
Glyma.05G086000.1.p	GmNAC160	177	11–140	1.5E-48		V	SNAC-B
Glyma.05G108700.1.p	GmNAC161	212	18–98	6.4E-9		V	ANAC001
Glyma.06G014900.1.p	GmNAC162	374	22–149	1.6E-49		VI	NAM
Glyma.06G288500.1.p	GmNAC163	285	49–189	3.5E-30		VI	ANAC001
Glyma.07G04790.1.p^**^	GmNAC164	497	12–141	2.1E-44	470–492	VII	unnamed
Glyma.07G192900.1.p	GmNAC165	362	15–142	3.8E-51		VII	SNAC-B
Glyma.08G163100.1.p	GmNAC166	348	6–133	1.8E-54		VIII	SNAC-B
Glyma.10G077000.1.p	GmNAC167	128	11–128	1.3E-38		X	SNAC-B
Glyma.10G077400.1.p	GmNAC168	136	11–136	1.7E-43		X	SNAC-B
Glyma.10G197600.1.p	GmNAC169	448	5–97	2.1E-29	424–446	X	NAM
Glyma.10G204700.1.p	GmNAC170	422	56–195	1.5E-28		X	ANAC063
Glyma.12G003200.1.p	GmNAC171	356	9–138	3.2E-51		XII	VND-NAC
Glyma.12G145100.1.p	GmNAC172	180	11–138	1.6E-46		XII	SNAC-B
Glyma.12G160100.1.p	GmNAC173	133	2–115	5.2E-35		XII	Senu5
Glyma.12G186900.1.p	GmNAC174	493	17–143	8.8E-47	303–325	XII	OsNAC8
Glyma.13G294000.1.p	GmNAC175	279	48–188	6.1E-30		XIII	ANAC001
Glyma.14G140100.1.p	GmNAC176	373	27–154	4.8E-53		XIV	NAM
Glyma.16G016400.1.p	GmNAC177	267	11–140	1.0E-44		XVI	unnamed
Glyma.16G069300.1.p	GmNAC178	399	56–198	4.8E-27		XVI	ANAC063
Glyma.18G301500.1.p	GmNAC179	229	14–138	1.3E-49		XVIII	Senu5
Glyma.19G002900.1.p	GmNAC180	389	56–197	1.9E-28		XIX	ANAC063
Glyma.19G108800.1.p	GmNAC181	336	8–138	5.8E-51		XIX	SNAC-B
Glyma.19G165600.1.p	GmNAC182	294	10–138	4.1E-48		XIX	NAM
Glyma.19G195800.1.p	GmNAC183	254	4–134	7.5E-43		XIX	TERN
Glyma.20G175500.1.p	GmNAC184	341	19–147	1.6E-51		XX	VND-NAC

* Orange indicates truncated NAC proteins harboring just a full-length NAC domain.

** Purple denotes NAC proteins with a transmembrane segment.

*** *Transmembrane segment*.

Among the 32 new genes identified, the genome analysis revealed 5 truncated NAC proteins (GmNAC158, GmNAC160, GmNAC167, GmNAC168, and GmNAC173), which harbor a complete NAC domain with the conserved sub-domains A–E (Olsen et al., 2005), but lack the C-terminus transactivation domain (Supplementary Table 1orange highlights). The presence of a full-length NAC domain in all new members was confirmed by multiple sequence alignment with the previously characterized GmNAC030 as a prototype (Supplementary Figure 1; Mendes et al., 2013). Most likely, these truncated GmNAC proteins represent either pseudogenes or transcriptional repressors, which retain the capacity to bind to promoters but are incapable of activating transcription. The previously described Gm*NAC089, GmNAC096, GmNAC108*, and *GmNAC119* genes were not found in the last version of the soybean genome (Supplementary Table 1-green highlights). According to the Phytozome's information (available in https://phytozome.jgi.doe.gov/pz/portal.html#!info?alias=Org_Gmax) the gene maintenance in a novel genome assembly requires that the coding nucleotide sequence (CDS) and deduced amino acid sequence share more than 90% sequence identity with the previous annotation. As a result, the NAC soybean superfamily was updated to 180 members, 148 already described and 32 novel members from this work.

**Figure 2:**
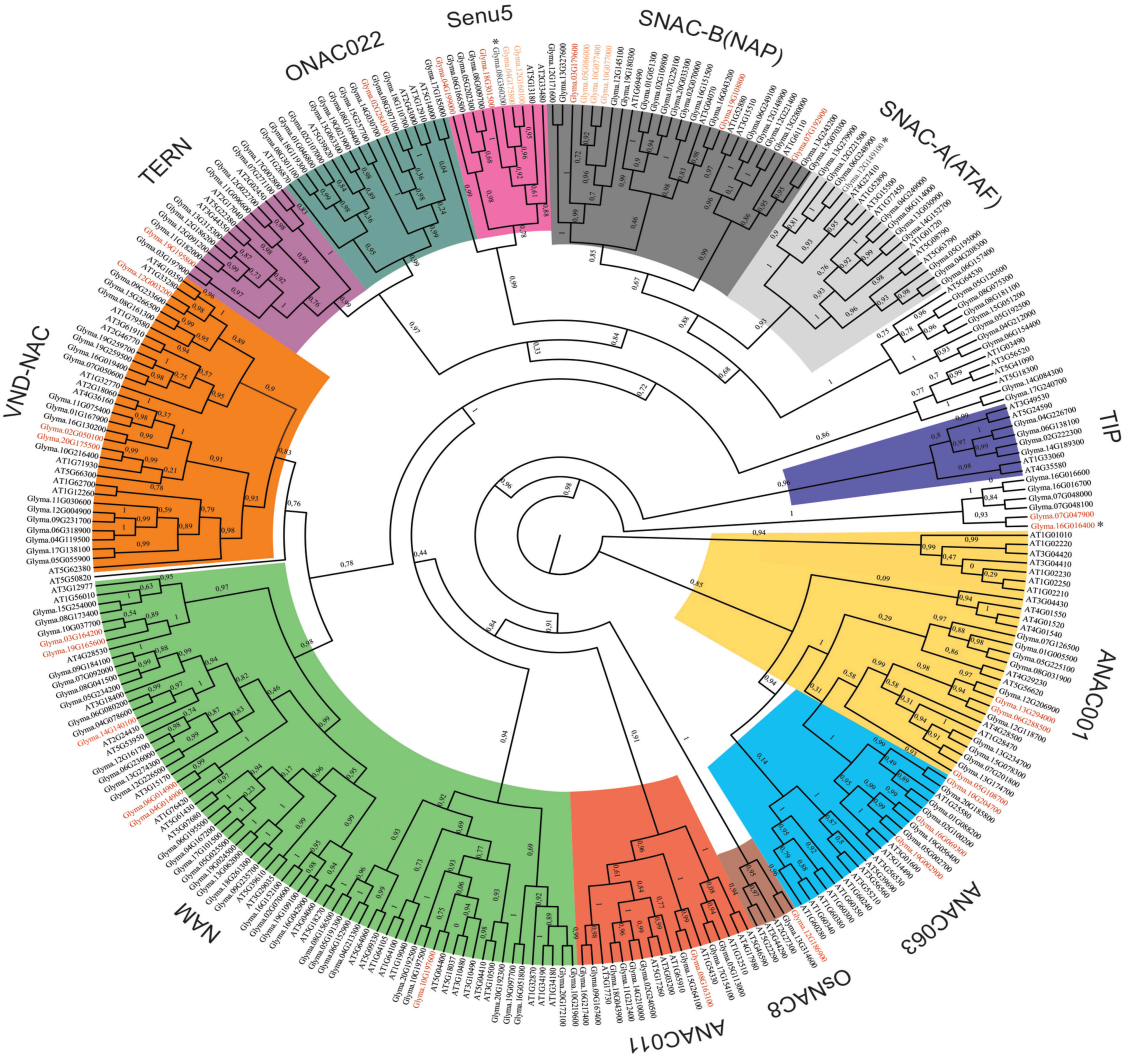
Phylogenetic reconstruction of the soybean and *Arabidopsis thaliana* NAC superfamily. All NAC deduced amino acid sequences from the soybean genome and the A*rabidopsis thaliana* genome were used to perform the phylogenetic analysis. Phylogenetic tree reconstruction was performed using the maximum likelihood statistical method with 10.000 bootstraps. NAC genes were grouped into 15 subgroups, including SNAC-A (ATAF—light gray), SNAC-B (NAP—dark gray), Senu5 (pink), ONAC022 (dark green), TERN (purple), VND-NAC (*vascular related NAC-*domain - orange), NAM (*no apical meristem* - light green), ANAC011 (red), OsNAC8 (brown), ANAC063 (light blue), ANAC001 (yellow), TIP *(turnip crinkle virus interaction protein* - dark blue) and unnamed groups (white). New putative NAC genes are shown in red. Asterisks indicate genes that were further characterized. Some possible pseudo-genes are shown in orange. The bootstrap score to each phylogenetic relation is shown at nodes.

The inventory of the soybean NAC superfamily from Le et al. (2011) demonstrated the presence of 11 transmembrane domain-containing transcription factors, which was later updated by Li et al. (2016b) to 15 putative membrane-tethered GmNAC proteins (NTL), including 3 novel identified ones and the GmNAC079, designated by Li et al. (2016b) as NTL8. Except for NTL5 whose pair (Glyma.16G016400) does not have a transmembrane (TM) segment, all the other 14 membrane-bound NACs are phylogenetically clustered in pairs and both duplicated genes have TM segments. This observation was reproduced in our phylogenetic analysis, which included the NAC genes from Arabidopsis (Figure 2).

The current phylogenetic analysis clustered the soybean NAC proteins into 15 subgroups with a high bootstrap score (>80%). This level of internal stability supported the classification of the soybean NAC superfamily into 15 subfamilies. The only exception was the ANAC063 subfamily that replicated with a low bootstrap score (0.14). Among them, 12 subfamilies were named according to previously characterized NAC clusters from Arabidopsis and rice and three subfamilies were unnamed because no reference of function or expression from these GmNACs was available (Ooka et al., 2003). The largest subfamily, NAM, comprises 23% of the NAC genes, followed by SNAC-B/NAP (12.8%) and VND-NAC (11.7%). The two least subfamilies were TIP (2.2%) and OsNAC8 (1.1%).

**Figure 3:**
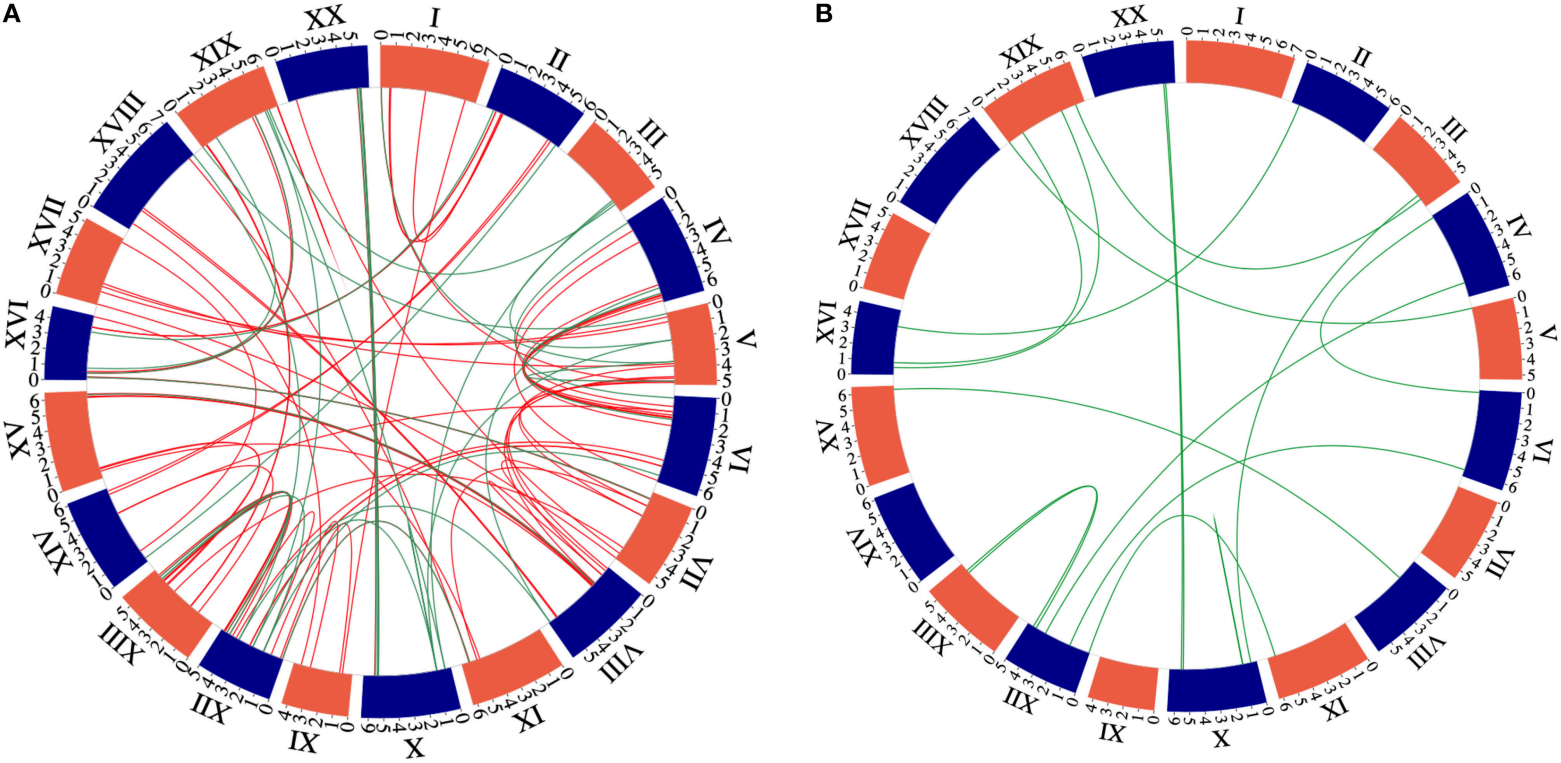
Circle plot of the soybean chromosomes and the GmNAC family. A. Circle plot of the soybean chromosomes showing the location of the new 32 NACs genes compared with the former NAC group. The green color represents 21 new NAC genes presented at the pairs of paralogous comparing with all NAC genes. We used the criterion of 80% identity to recover the paralogous pairs. B. Circle plot of soybean chromosomes and the 21 newly identified NAC genes displayed as duplicated gene pairs based on the phylogenetic analysis.

The 32 newly discovered GmNAC genes were distributed phylogenetically among the soybean NAC subfamilies and the soybean chromosomes (Figure 1, in red and Figure 2, in red and orange). The new mapped genes did not change the density of GmNACs among the 20 soybean chromosomes (chromosome 12 kept the highest number of GmNACs, 9.4%; Supplementary Figure 2) with an increment of three possible tandem duplications (in chromosomes X, XII, and XVI; Figure 1), which were not confirmed by the phylogenetic and synteny analyses (see *GmNAC174*, Glyma.12G186900, OsNAC8 subfamily and *GmNAC177*, Glyma.16G016400, unnamed subfamily; Figures 2, 3 and Table 2). For the new genes, the only possible tandem duplication confirmed by the synteny analysis refers to the pair *GmNAC167* (Glyma.10G077000) and *GmNAC168* (Glyma.10G077400) in chromosome X, SNAC-B(NAP) subfamily (Figure 3B). However, the inclusion of the new NAC proteins in our phylogenetic analysis showed that 21 of 32 new NAC genes were clustered in pairs, consistent with duplication events. The synteny-based analysis focused on the distribution of the new 32 genes on the genome (Figure 3A) and in the 21 duplicated pairs containing new NAC genes (Figure 3B and Table 2). The significant number of new NAC genes duplications had spread among the chromosome. A few syntenic groups have found at the chromosome X and XX as well as XII and XIII (Figure 3A). A striking feature of the soybean genome is the retention of extended blocks of duplicated genes with a low frequency for reversion to singletons (Schlueter et al., 2008). Concerning the membrane-tethered GmNAC (NTL) transcriptional factors, 14 of 15 GmNTLs are clustered as paralogs (Li et al., 2016b). An evolutionary and functional analysis of the soybean NTL genes, previously described, indicated that the duplicated genes diverge functionally, which contributes as an evolutionary driving force for the retention of these GmNTL duplicate genes (Li et al., 2016b). This precedent in the literature raised the possibility that other GmNAC pairs may exhibit partially overlapping but distinct functions.

**Table 2:** Divergence between NAC gene pairs in soybean.

							**Duplication**		
Gene Name^*^	**Gene Name**	**Ka**	**SD**	**Ks**	***SD***	**Ka/Ks**	Date (Mya)^**^	**Selection**	*p*-value (FDR)^***^
Glyma.07G047900 (GmNAC164)	Glyma.16G016400 (GmNAC177)	0.05994566	0.0144061	0.1303571	0.0363278	0.459857269	10.69	Purifying	0.03557996
Glyma.12G206900 (GmNAC090)	Glyma.13G294000 (GmNAC175)	0.01789244	0.00727299	0.1173597	0.0337963	0.152458127	9.62	Purifying	0.05012003
Glyma.06G288500 (GmNAC163)	Glyma.12G118700 (GmNAC083)	0.01752367	0.00879841	0.2001884	0.0489479	0.087535891	16.41	Purifying	0.08495689
Glyma.10G204700 (GmNAC170)	Glyma.20G185800 (GmNAC150)	0.02990029	0.00760776	0.06833386	0.01774	0.437561847	5.60	Purifying	0.02610727
Glyma.16G069300 (GmNAC178)	Glyma.19G056400 (GmNAC142)	0.04890274	0.0105635	0.0998735	0.0245792	0.489646803	8.19	Purifying	0.02745994
Glyma.05G002700 (GmNAC025)	Glyma.19G002900 (GmNAC180)	0.05709798	0.0122494	0.08683529	0.0248523	0.657543494	7.12	Purifying	0.02610727
Glyma.12G186900 (GmNAC174)	Glyma.13G314600 (GmNAC103)	0.07831763	0.014255385	0.191407	0.040780976	0.409168056	15.69	Purifying	0.0429662
Glyma.08G163100 (GmNAC166)	Glyma.15G264100 (GmNAC117)	0.02198773	0.0070702	0.08570832	0.0238959	0.256541372	7.03	Purifying	0.03736929
Glyma.06G014900 (GmNAC162)	Glyma.04G014900 (GmNAC157)	0.05260988	0.011301031	0.07860409	0.024974149	0.669302068	6.44	Purifying	0.02610727
Glyma.03G164200 (GmNAC155)	Glyma.10G037700 (GmNAC071)	0.1584804	0.025821503	0.7327278	0.153642442	0.216288231	60.06	Purifying	0.20150153
Glyma.10G216400 (GmNAC073)	Glyma.20G175500 (GmNAC184)	0.02807372	0.00854546	0.1685033	0.0449916	0.166606351	13.81	Purifying	0.0644157
Glyma.02G050100 (GmNAC153)	Glyma.16G130200 (GmNAC126)	0.03756366	0.00954128	0.07316453	0.0223967	0.513413535	6.00	Purifying	0.02610727
Glyma.09G233600 (GmNAC069)	Glyma.12G003200 (GmNAC171)	0.007916301	0.0046751	0.06380496	0.0233777	0.124070307	5.23	Purifying	0.03736929
Glyma.03G197900 (GmNAC014)	Glyma.19G195800 (GmNAC183)	0.06498027	0.0164195	0.1111914	0.0379041	0.584400142	9.11	Purifying	0.02610727
Glyma.12G160100 (GmNAC173)	Glyma.04G175800 (GmNAC158)	0.00671151	0.0067342	0.02543455	0.01863	0.263873747	2.08	Purifying	0.02610727
Glyma.10G077000 (GmNAC167)	Glyma.10G077400 (GmNAC168)	0.0410012	0.0166212	0.02319112	0.0136607	1.767969809	1.90	Positive	0.02610727
Glyma.16G043200 (GmNAC124)	Glyma.19G108800 (GmNAC181)	0.0346605	0.0101229	0.1196774	0.0345572	0.289616085	9.81	Purifying	0.04214007

* The 21 duplicated genes retrieved from the gourp of the 32 new NACs are shown in red.

** Based on a rate of 6.1 × 10^−9^ substitutions per site per year, it was calculated the divergenece time (T) as T = Ks/(2 × 6.1 × 10^−9^) x 10^6^ Mya (Zhang et al., 2018).

*** *H0: Ka = Ks; The P-value corrected by false discover rate (FDR) was calculated based on multiple chi-square test*.

The soybean genome has undergone two duplication events, which occurred approximately 59 and 13 million years ago (Schmutz et al., 2010). As for the 21 duplicated gene pairs containing new NAC genes, except for 3 NAC gene pairs with a divergence period higher than 13 Mya, the remaining 18 duplicated gene pairs were formed by the second event of whole genome duplication of *Glycine max* (Table 2). The molecular evolution of the new NAC duplicated pairs were investigated by calculating the ratio of non-synonymous to synonymous substitutions (Ka/Ks). Only the duplicated gene pair Glyma.10G077000 (*GmNAC167*) / Glyma.10G077400 (*GmNAC168*) with a significant ratio Ka/Ks > 1 may have undergone a positive selection in soybean. The other 20 duplicated gene pairs had been under purifying selection in soybean, as suggested by their Ka/Ks ratio <1.

#### Organ- and Tissue-Specific Expression Profile of the 32 New NAC Genes

**Figure 4:**
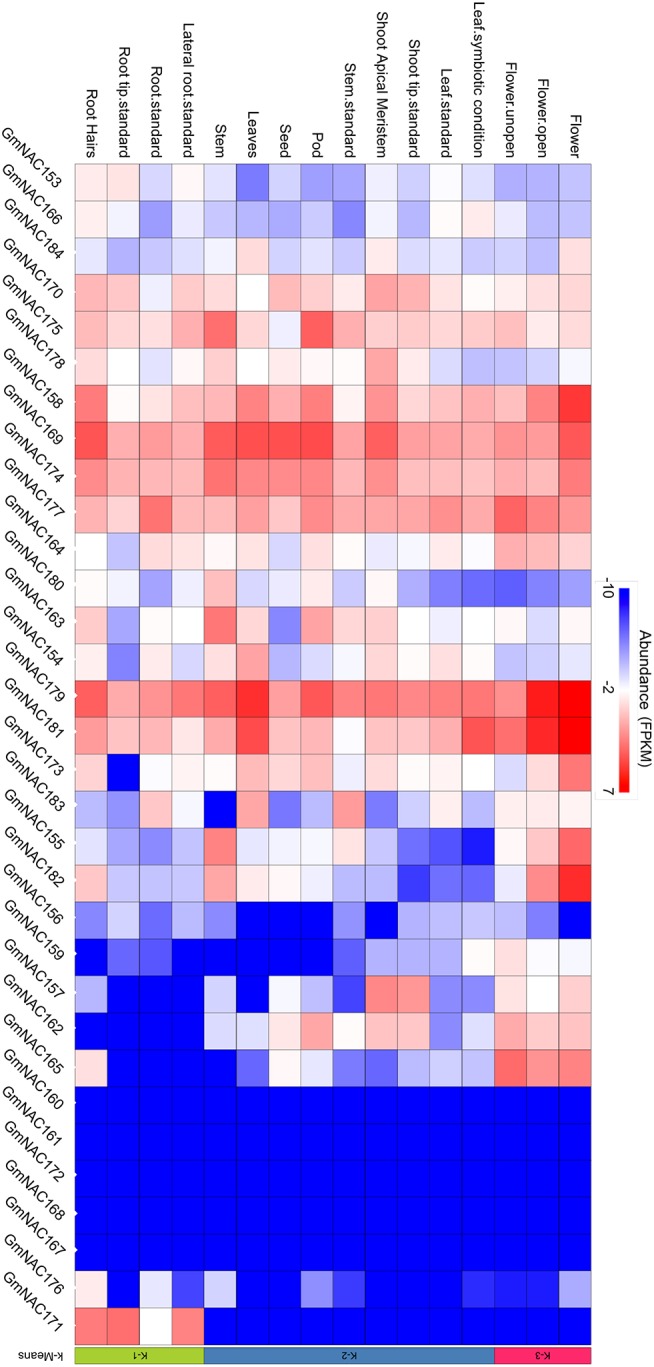
Organ and tissue-specific expression of the new NAC genes. The heat map plot was created by the PhytoMine tool (https://phytozome.jgi.doe.gov/phytomine/begin.do) presented at the Phytozome v12 website (https://phytozome.jgi.doe.gov). The heat map was built using all gene expression data at the database regarding tissue- and organ-specific expression.

**Figure 5:**
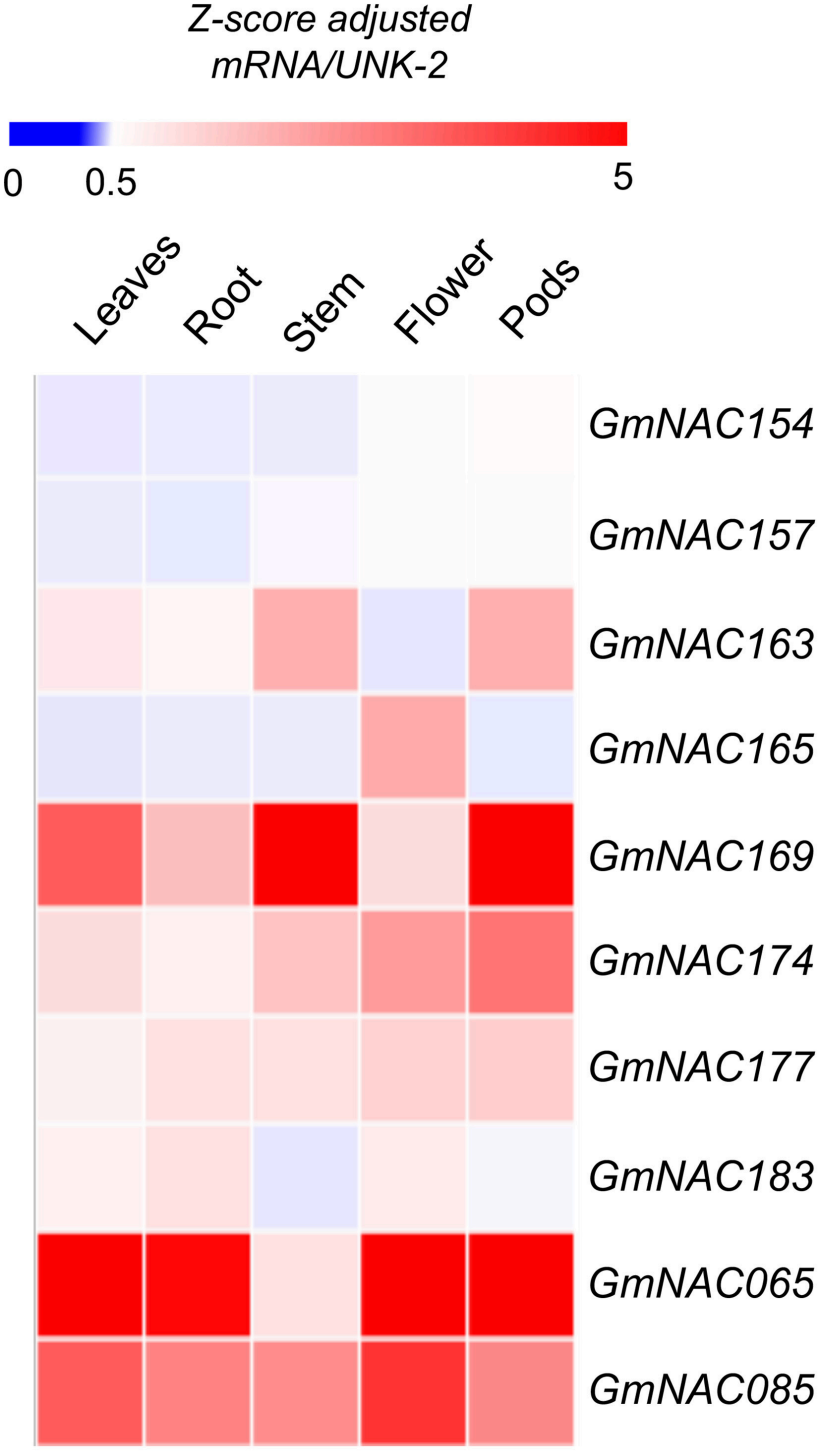
GmNAC gene expression profile in different soybean tissues. Heat map of 8 putative NAC genes in addition to GmNAC065 and GmNAC85. The expression levels of a randomly selected representative sample of new GmNAC genes were measured in R2/R3 plants under normal growth conditions by qRT-PCR. The *UNK-2* gene was used as endogenous control. The data displayed in the figure comprise the qRT-PCR from Supplementary Figure 3 and Supplementary Table 5.

The NAC genes are involved in developmental programs and stress-response. To gain insights into potential developmental roles of the new NAC genes and to validate these new genes in the soybean genome, we analyzed the expression profile of these genes in different developmental stages and tissues based on publically accessed transcriptomes obtained from phytozome (Figure 4). These data were confirmed by qRT-PCR of a sub-set of NAC genes (Figure 5, Supplementary Figure 3 and Supplementary Table 5). All the newly identified NAG genes are expressed in some, but not all, tissues. A group of new NAC genes is highly expressed in most tissues, including *GmNAC158, GmNAC169, GmNAC174, GmNAC177, GmNAC179*, and *GmNAC181*, which was confirmed by qRT-PCR for *GmNAC169, GmNAC174*, and *GmNAC177* (Figure 5). There is also a group of newly identified GmNACs, which displays low transcript accumulation in all tissues analyzed (see the dark blue cluster of genes). Some genes displayed a distinct tissue-specific expression, suggesting different functions in development. While *GmNAC153* and *GmNAC166* are most expressed in roots, a sub-set of genes (*GmNAC155, GmNAC165*, and *GmNAC182*) is highly expressed in flowers, for example. Quantitative RT-PCR confirmed that the GmNAC165 transcript accumulates to high levels in flower (Figure 5 and Supplementary Figure 3).

Among the 32 new NAC genes, we observed 3 duplicated gene pairs containing only new NAC genes (Table 2). Except for the *GmNAC167*/*GmNAC168* pair which had undergone positive selection (Table 2), the other two pairs *GmNAC162*/*GmNAC175* and *GmNAC173*/*GmNAC158*, which had experienced purifying selection, exhibited distinct expression patterns in different tissues. This profile may suggest diversification in expression level, which may be associated with a certain degree of sub-functionalization as the mechanism for the retention of these duplicated GmNAC genes.

### Stress Regulation of a Representative Sample of the Newly Identified GmNAC Genes

**Figure 6:**
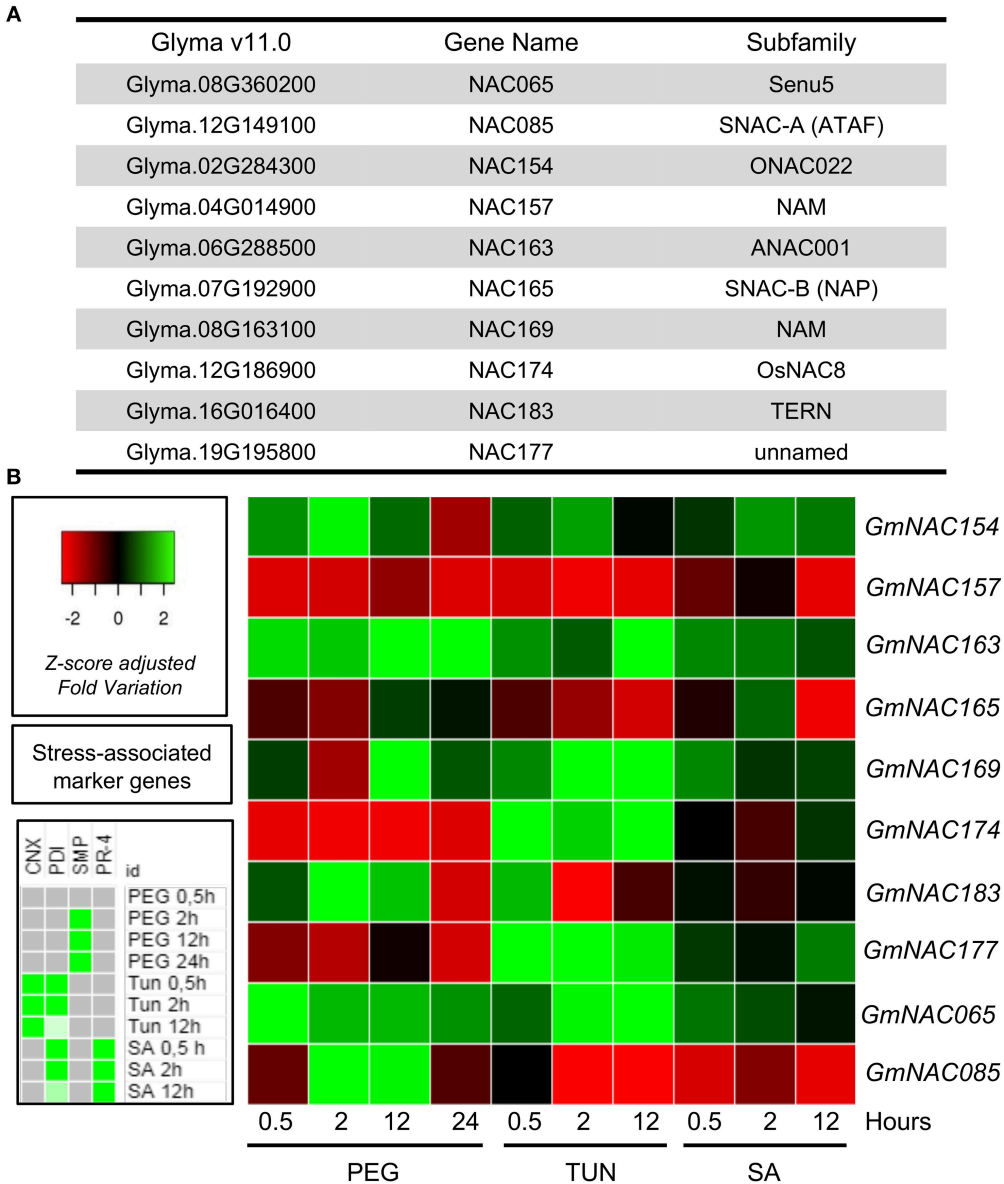
Expression patterns of new GmNACs during multiple stress. **(A)** The identity of the selected GmNAC genes. **(B)** Heatmap of the stress-induced expression of 8 new putative GmNAC genes, GmNAC065 and GmNAC85. For the expression analysis of randomly selected GmNACs, soybean seedlings were stressed with PEG (10%), tunicamycin (5 μg/mL) and salicylic acid (5 mM) for 0.5, 4 and 12 h (24 h, exclusively for PEG treatment) and the transcript accumulation was monitored by qRT-PCR. The *UNK-2* gene was used as endogenous control, and gene-associated stress markers were used to monitor the effectiveness of treatment (on the left). The data presented in the figure comprise the qRT-PCR from Supplementary Figures 4, 5 and Supplementary Table 6.

Comparing Arabidopsis and soybean, several GmNAC subfamilies have undergone expansion after their speciation. The domestication process of soybean could account at least in part for this variation because some GmNACs have been linked to traits targeted by artificial selection such as drought tolerance and morphological adaptation (Dong et al., 2013; Thao et al., 2013; Thu et al., 2014; Hussain et al., 2017). These data suggest that the expansion of soybean NAC genes may reflect amplification or lineage-specific responses to various biotic and abiotic stress responses under the influence of artificial selection. In view of this observation, we next examined the stress-induced expression profile for a representative sample (eight genes) of the new NAC genes (Figure 6A). This quantitative expression analysis also allowed us to validate further these new genes in the soybean genome. Three-week-old soybean seedlings were treated with the osmotic-stress inducer PEG, the ER-stress inducer TUN, and the defense inducer SA. Three-period intervals after the treatment were chosen (0.5, 2, 12, and 24 h to PEG) to include a time range of early responses, intermediate responses, and late responses. We also examined the expression of the osmotic stress-induced control gene *SMP*, ER stress-induced control genes*, CNX* and *PDI*, and the SA-induced control *PR1* to monitor the effectiveness of the respective stress treatment (Supplementary Figure 4). All examined GmNACs were regulated by at least one of the stress treatments, confirming they are expressed in response to some stimulus (Figure 6B).

*GmNAC154, GmNAC163, GmNAC169*, and *GmNAC065* were up-regulated by PEG, TUN and SA, although to different extents and kinetics of induction (Figure 6, Supplementary Figure 5 and Supplementary Table 6). In contrast, *GmNAC157* was down-regulated by all stress treatments. The four up-regulated genes belong to subfamilies with stress-responsive members (Figure 6A), whereas *GmNAC157* is most related to AT1G76420 (*ANAC031*), also designated CUP-SHAPED COTYLEDON3 (*CUC3*), which has not been implicated in stress responses. It functions partially redundant with *CUC1* and *CUC2* in the establishment of the cotyledon boundary specification and the shoot meristem (Hibara et al., 2003; Vroemen et al., 2003).

The expression profile of *GmNAC174* and *GmNAC177* was similar, down-regulated by PEG, but up-regulated by TUN and SA, which was in marked contrast with *GmNAC085*. While *GmNAC085* belongs to SNAC-A (ATAF) subfamily, which harbors stress-responsive members, *GmNAC174* is closely related to the ER stress-induced *ANAC089* (AT2G22290) and *GmNAC177* clusters closely to the TIP subfamily with members involved in biotic interactions and immune response control. Therefore, they display a stress-responsive expression profile consistent with their subfamily classification.

*GmNAC165* was repressed by prolonged ER stress and SA treatment, which is consistent with its classification as a SNAC-B (NAP) member. GmNAC183, which is a member of the TERN subfamily, was rapidly (0.5 h) induced by PEG and TUN but repressed if the stress persisted. The members of the TERN subfamily GmNAC081 and ANAC36 has been shown to be induced by PEG and TUN (Reis et al., 2011, 2016; Mendes et al., 2013). Therefore, the stress-responsive expression profile of the novel GmNACs is consistent with their phylogenetic relatedness as they share similar expression profile with homologous genes of the soybean or other plant species that belong to the same subfamily.

### Genome-Wide Expression Analysis Uncovers an Extended List of Senescence-Associated GmNACs

**Figure 7:**
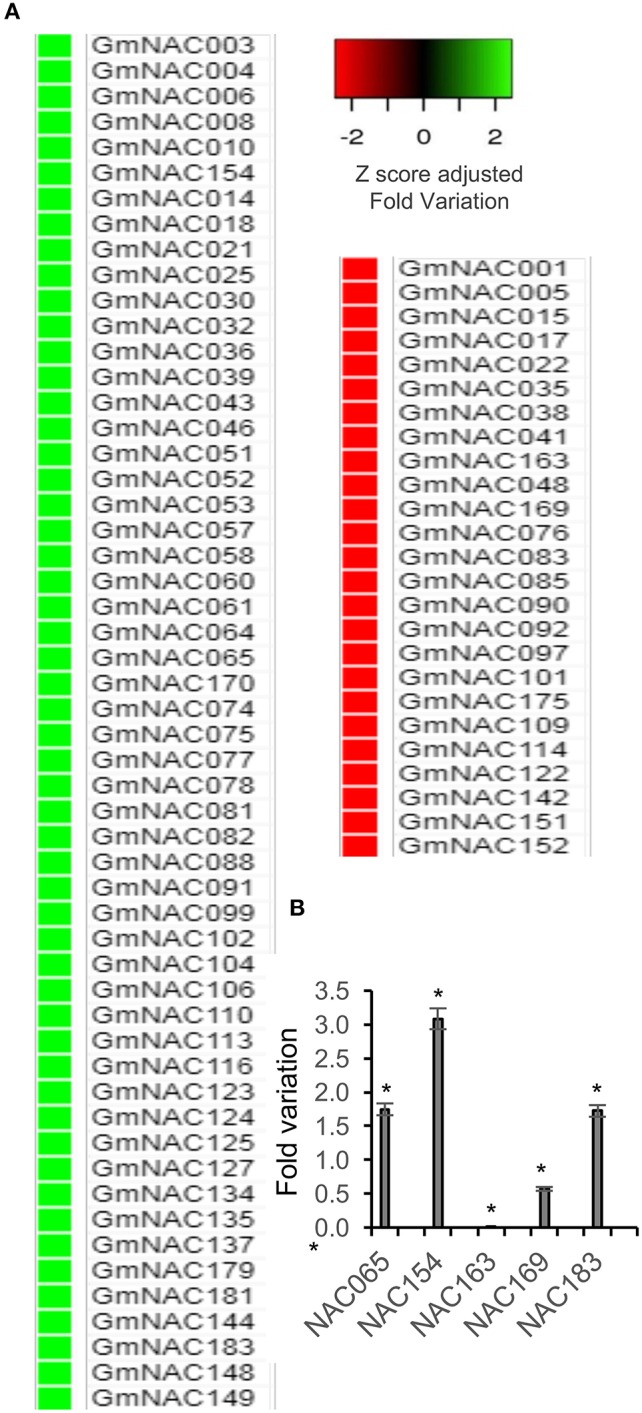
Expression profile of NAC genes during natural leaf senescence in soybean. **(A)** Heatmap of senescence-associated GmNAC genes. The data presented in the figure comprise an RNA-seq analysis showing the differential expression of NAC genes in BR16_80d (R7)-BR16_20d(V3). **(B)** Senescence-induced variation of expression for representative senescence-associated NAC genes. Total RNA was isolated from 20 DAG to 80 DAG soybean leaves and the transcript accumulation of the indicated genes was measured by qRT-PCR. *UKN-2* was used as the normalizer, endogenous control gene. Relative gene expression was quantified using the comparative 2^−ΔΔCt^ method. The bars indicate standard-error and the asterisks indicate statistical significance by the *t*-test, (*P* < 0.05, *n* = 3).

High-resolution temporal expression profiles associate approximately one-third of Arabidopsis NAC genes with senescence (Breeze et al., 2011) and functional studies of a few members have been conducted confirming their crucial role in developmentally programmed senescence (Hickman et al., 2013; Kim et al., 2018). In addition to identifying new NAC genes in the soybean genome, we performed a transcriptomic analysis to examine the differential expression of GmNACs during natural leaf senescence. Remarkably, 44% of the GmNAC genes were differentially expressed (DE) at the onset of senescence [BR17_80d(R7)-BR16_20d(V3)], including eight (8) newly identified GmNACs (Figure 7, Supplementary Table 7). In this set of senescence DE GmNAC genes, the up-regulated changes predominated over the down-regulated changes (Figure 7A). Among the up-regulated genes, GmNAC030 (SNAC-A/ATAF subfamily) and GmNAC081 (TERN subfamily) have already been demonstrated to be induced by leaf senescence (Carvalho et al., 2014b) and GmNAC081 functions as a positive regulator of developmentally programmed leaf senescence (Pimenta et al., 2016). The results of RNA-seq were further confirmed by qRT-PCR targeting a representative sample of senescence-induced and senescence-repressed genes (Figure 7B, Supplementary Table 9). During senescence, *GmNAC065, GMNAC154* and *GmNAC183* were induced, whereas *GmNAC163*, and *GmNAC169* were down-regulated as demonstrated by RNA-seq.

The most represented subfamily of senescence-associated genes was the SNAC-A (ATAF) family (90%) followed by TERN (80%) and TIP (75%). No senescence-associated NAC gene was detected in subfamilies OsNAC8 and ANAC011 (Supplementary Table 8). All the senescence DE genes from subfamilies SNAC-B (NAP), TERN, TIP, and Senu5 were up-regulated during senescence. Accordingly, the representative type of the SNAC-B(NAP) subfamily, AtNAP, has been shown to function as a positive regulator of leaf senescence (Guo and Gan, 2006) and representatives of the TERN subfamily, like GmNAC081, have also been shown to control leaf senescence (Pimenta et al., 2016).

### Biological and Biochemical Properties of Previously Uncharacterized GmNAC Genes

**Figure 8:**
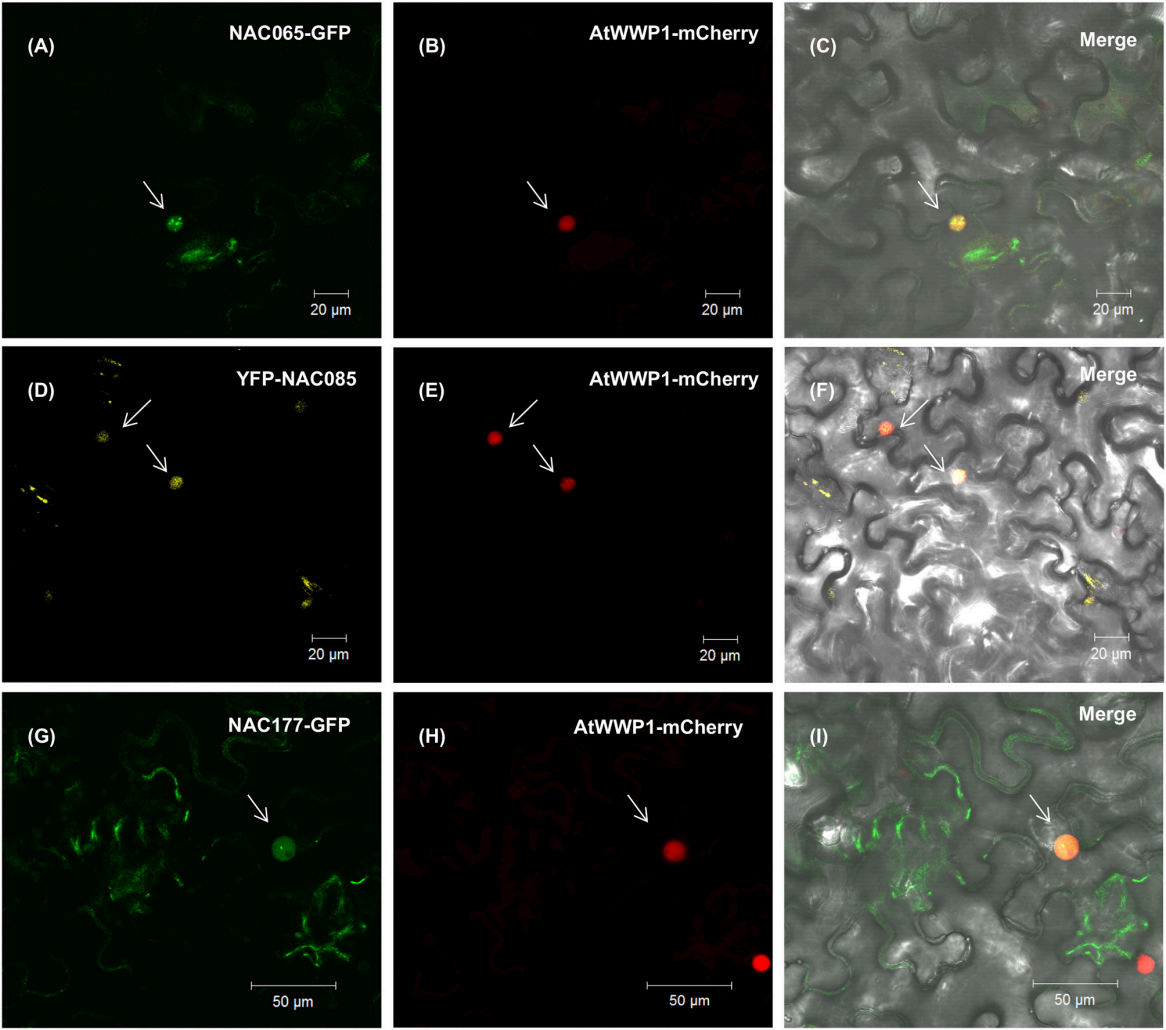
NAC065, NAC085, and NAC177 subcellular localization. Leaves of *Nicothiana benthamiana* were infiltrated with *Agrobacterium tumefaciens* GV3101 carrying the GFP-NAC or YFP-NAC constructs, and the localization of the fused protein was monitored by confocal microscopy. In the left column, **(A,D,G)** show the expression of NAC065-GFP, YFP-NAC085 and NAC177-GFP (top to bottom) in the nuclei, indicated by white arrows. In the middle column, **(B,E,H)** show the expression of the nuclear marker AtWWP1-mCerry in the corresponding nuclei field of the first column. **(C,F,I)** merged images of NAC fusions and AtWWP1 marker in the bright-field.

Based on expression profiles and phylogenetic relatedness between already characterized *A. thaliana* NACs and the homologous soybean genes, one can formulate a hypothesis about their precise functions. We examined next the biochemical and biological properties of two uncharacterized NAC genes, *GmNAC065* (Glyma.08G360200; Senu5) and *GmNAC085* [Glyma.12G149100; SNAC-A (ATAF)], in addition to *GmNAC177* (Glyma.16G016400; unnamed subfamily) as a representative of the novel NAC genes. We showed first that all three NAC genes are nuclear localized, as the fluorescence of transiently expressed GFP-fused NAC genes (GmNAC065-GFP, GmNAC085-GFP, and GmNAC177-GFP) concentrated in the nucleus and co-localized with a nuclear marker gene, which was fused to mCherry (Figure 8; Calil et al., 2018). The fluorescence from the expression of GFP alone displayed a different pattern and concentrated in the cytosol and nucleus (Supplementary Figure 6).

**Figure 9:**
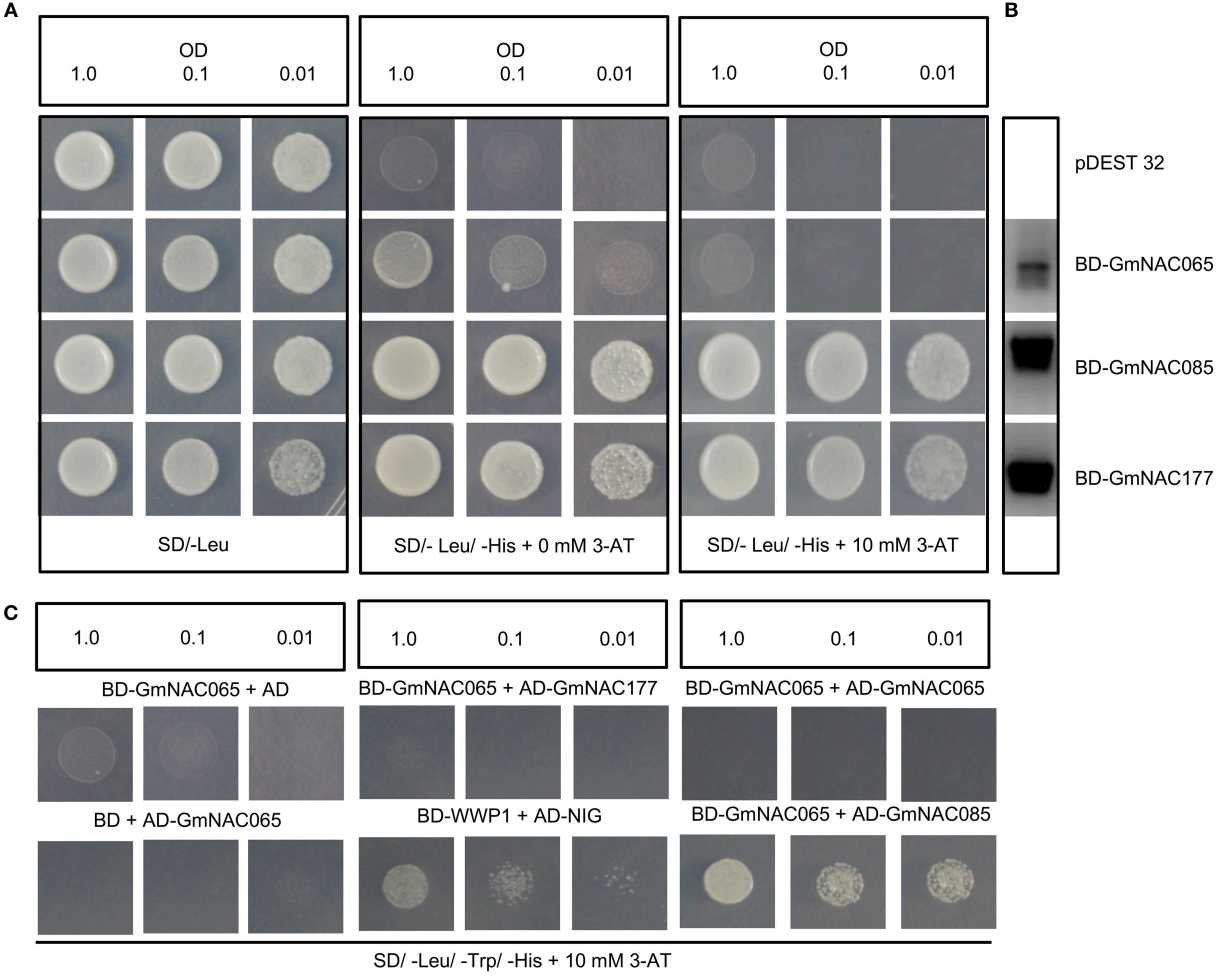
NAC065, NAC085 and NAC177 transactivation capacity and protein-protein interaction. **(A)** Transactivation activity of GmNAC proteins. The fusions BD-NAC065, BD-NAC085 and BD-NAC177, and the empty vector were transformed separately into yeast strains AH109 and activity was determined by monitoring His prototrophy on selective medium. The transformants were incubated for 3 days at 28°C in SD media lacking leucine and histidine but supplemented with 10 mM 3-aminotriazol (3AT). **(B)** Expression analysis of BD-NAC065, BD-NAC085 and BD-NAC177 fusions in yeast. Total RNA was extracted from yeast cells transformed with the indicated DNA constructs, and gene expression was monitored by RT-PCR. **(C)** Interactions of GmNAC065 with GmNAC085 in yeast. GmNAC065 was expressed in yeast as GAL4 binding domain (BD) fusion, and GmNAC085 and GmNAC177 were expressed in yeast as GAL4 activation domain (AD) fusions. Interactions between the tested proteins were examined by monitoring His prototrophy in the presence of 10 mM 3-AT. In the control experiments, the GmNAC065 fusions was expressed with the reciprocal empty vector (pBD or pAD).

To investigate whether GmNAC065, GmNAC085, and GmnAC177 harbor a functional transcriptional activator domain, we used a yeast transactivation assay, in which the coding region of each *GmNAC* gene was fused to the DNA binding domain of GAL4 (BD) and *S. cerevisiae* AH109, harboring a *HIS3* reporter gene under the control of GAL4 promoter, was transformed with the BD-NAC fusions (Figure 9). The expression of the BD-NAC fused genes in yeast was monitored by RT-PCR (Figure 9B). Then, we monitored the capacity of the fusion proteins (BD-GmNACs) to activate transcription of *HIS3* reporter gene and hence to display His prototrophy (Figure 9A). The yeast transforming lines expressing BD-GmNAC085 and BD-GmNAC177 fusions displayed the most pronounced growth in the selective medium SD-Leu-His, supplemented with 10 mM 3-AT. The BD-NAC065 lines displayed a slow growth rate in histidine depleted medium, but growth was prevented in medium supplemented with 10 mM 3-AT. These results demonstrated that GmNAC085 and GmNAC177 display transactivation activity in yeast, which was in contrast with GmNAC065 that may act as a transcriptional repressor. Several precedents in the literature have evoked a repressive transcriptional function of NAC proteins from different species (Delessert et al., 2005; Lu et al., 2007). Alternatively, GmNAC065 may depend on specific interactions with other transfactors to activate their target genes as heterodimers. The lack of transactivation activity in yeast has also been observed for GmNAC081, which belongs to the subfamily TERN, and interacts with GmNAC030 from the SNAC-A (ATAF) subfamily to activate the expression of *VPE* (vacuolar processing enzyme) that promotes cell death (Pinheiro et al., 2009).

We then asked whether GmNAC065 (Senu5 subfamily) would form a homodimer and/or interact with GmNAC085 (SNAC-A/ATAF subfamily) or GmNAC0177 (unnamed subfamily) through yeast two-hybrid assays. While GmNAC065 was fused to the GFAL4-BD, GmNAC085, and GmNAC177 were fused to the GAL4-AD (Figure 9C). The coexpression of the BD-GmNAC065 and AD-GmNAC085 fusion proteins promoted the growth of yeast in the selective medium, which was more pronounced than that of the positive control (BD-WWP1+AD-NIG), whereas the coexpression of BD-GmNAC065 with either AD-GmNAC065, AD-GmNAC177, or pAD did not promote his prototrophy. These results confirmed the specific interaction of GmNAC065 with GmNAC085 and raised the possibility that GmNAC065 may function as heterodimers in cell death events, as *GmNAC065* is a member of Senu5 subfamily induced by leaf senescence (Figure 7) and *GmNAC085* is a member of the SNAC-A (ATAF) subfamily that harbors cell death-induced members (Figure 2). However, these putative partners are not coordinately expressed in response to stress and developmental programs. While *GmNAC065* is induced by developmentally programmed leaf senescence (Figure 7), PEG, TUN, and SA (Figure 6) and repressed by drought (Carvalho et al., 2014a), the expression of *GmNAC085* is repressed by senescence, TUN, SA and induced by PEG (Figures 6, 7) and drought (Carvalho et al., 2014a). Therefore, the only overlap in the expression profile of *GmNAC065* and *GmNAC085* is the up-regulation by PEG, which may indicate heterodimerization under this stress condition.

### The Involvement of GmNAC065, GmNAC085, and GmNAC177 With Cell Death Progression

**Figure 10:**
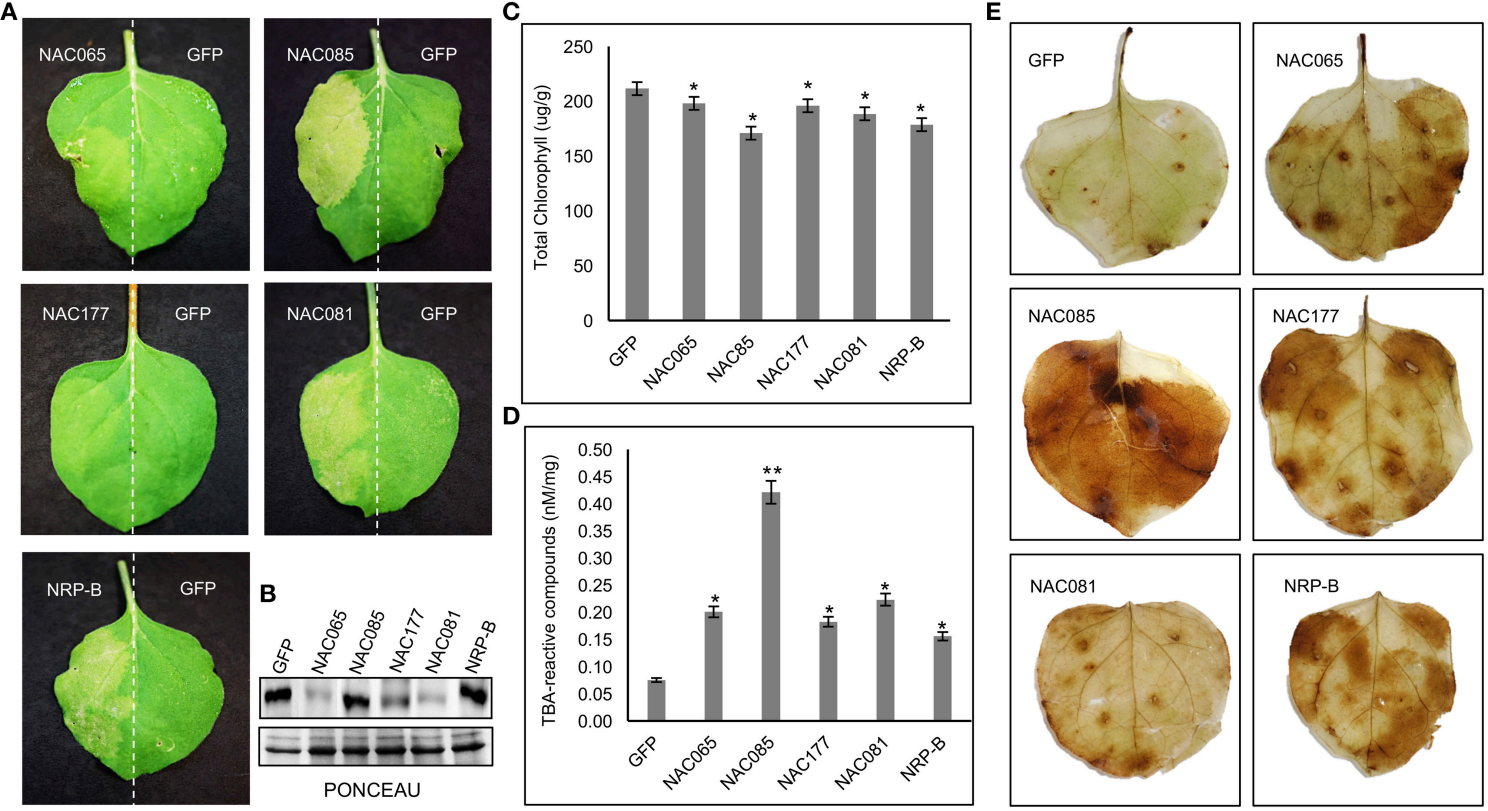
Transient expression of NAC065, NAC085, and NAC177 induces cell death *in planta*. **(A)** *Nicotiana benthamiana* leaves were agroinfiltrated with GmNAC065-GFP, GmNAC085-GFP or GmNAC177-GFP on one side and the other half of the leaves was agroinfiltrated with GFP (as a negative control). GmNAC081 and NRP-B were used as positive controls. **(B)** Accumulation of NAC proteins in agroinfiltrated leaf sectors. Total proteins were extracted from agroinfiltrated leaf sectors and immunoblotted with an anti-GFP antibody **(C)** Total chlorophyll content 3 days after agroinfiltration. **(D)** TBA-reactive compounds accumulation after NAC expression. **(E)** DAB leaf-staining 3 days after agroinfiltration. The bars indicate standard-error and the asterisks indicate statistical significance by the *t*-test: ^*^(*P* < 0.05, *n* = 3); ^**^(*P* < 0.01, *n* = 3).

To address the possibility that *GmNAC065, GmNAC085, and GmNAC177* are involved in cell death events, the transient expression of NAC genes was induced by agroinfiltration into *N. benthamiana* leaves and the resulting phenotypes were compared with those displayed by previously characterized senescence-associated genes, including *GmNAC081* and *NRP-B* (Faria et al., 2011; Reis et al., 2011; Figure 8A). GmNACs and controls accumulated stably in the agroinoculated leaf sectors (Figure 10B). After three days, the leaf sectors that were agroinoculated with the DNA constructs expressing *GmNAC065, GmNAC085, and GmNAC177* and the positive controls *NRP-B* and *GmNAC081* (Figure 10B) displayed a chlorotic phenotype characteristic of leaf senescence that was associated with decreased chlorophyll content and consequent yellowing of the leaf sector (Figures 10A,C). We further examined the GmNACs-induced senescence by measuring the accumulation of thiobarbituric acid (TBA)-reactive compounds such as malondialdehyde (MDA). These compounds are products of senescence-associated lipid peroxidation, a process resulting in the generation of reactive oxygen species. A significantly higher concentration of TBA-reactive compounds was observed in the *GmNAC065*-, *GmNAC085*-, *GmNAC177*-expressing leaf sectors and in the *NRP-B-* and *GmNAC081* control sectors than in the *GFP*-expressing leaf sectors (Figure 10D). Likewise, the accumulation of H_2_O_2_ was enhanced in GmNACs and *NRP-B* agroinoculated leaves in comparison with *GFP*-expressing leaves (Figure 10E). Collectively, our results indicate that transient expression of *GmNAC065, GmNAC085*, and *GmNAC177* in *N. benthamiana* leaves induces senescence and cell death. Because *GmNAC065* and *GmNAC085* are differentially expressed during leaf senescence they may be involved in developmentally programmed leaf senescence in soybean. In contrast, the newly identified *GmNAC177* was not differentially regulated during natural leaf senescence, but it may be involved in cell death events induced by biotic stress, as it clusters in an unnamed subfamily that is closely related to the TIP subfamily of GmNACs implicated in pathogen-host interactions.

## Discussion

### Phylogenetic Reconstruction of the GmNAC Superfamily Enlarges This Family to 180 Members Distributed Into 15 Structurally and Functionally Conserved Subfamilies

Despite the extensive literature regarding the plant-specific TFs of the NAC family, functional studies have been restricted to few members of the family as they have been conducted primarily with orthologs of different species, multiplying the number of GmNACs analyzed but with similar function. Because the extension and complexity of the NAC family in the plant kingdom have not been thoroughly examined, orthologous genes from different species cannot be fully predicted. Here we performed an in-depth examination of the NAC superfamily from soybean, interrogating the last version of the soybean genome, version Wm82.a2.v1-v11.0. The phylogenetic reconstruction of the GmNAC superfamily uncovered 32 new members in the soybean genome, raising the size of this family to 180 members. As compared to the Arabidopsis NAC superfamily, the soybean GmNAC subfamilies showed an extensive expansion in the number of their NAC members, which may be a result of artificial selection associated with relevant agronomic traits, including yield, drought tolerance, pathogen resistance, etc. As an important economic crop, soybean has been exposed to intense breeding programs, which may have contributed to the establishment of the current NAC superfamily profile in this species.

This comprehensive analysis comparing the complete repertory of Arabidopsis and soybean NACs provided a framework to rationalize future functional studies of the members of this family. In fact, in addition to sequence conservation, the previously characterized soybean NACs were correctly placed in the corresponding subfamily based on expression profile and function. Accordingly, GmNAC030 (Glyma.05G195000), which has been characterized as a component of the stress-induced NRP/DCD-mediated cell death signaling (Mendes et al., 2013), was clustered with members of the SNAC-A subfamily of stress-induced NAC proteins, which encompasses highly conserved and functionally characterized proteins from Arabidopsis, displaying partially overlapping function, such as ATAF1 (AT1G01720) and ATAF2 (AT5G08790). *ATAF1* has been shown to be induced by both biotic and abiotic stress, to regulate negatively drought stress-responsive genes and positively ABA biosynthesis (Lu et al., 2007; Wu et al., 2009; Jensen et al., 2013). *ATAF1* also serves as a positive regulator of senescence by coupling stress-induced response with photosynthesis- and senescence-related transcriptional cascade in addition to acting as a negative regulator of defense responses against both necrotrophic fungal and bacterial pathogens (Wang et al., 2009; Garapati et al., 2015). ATAF2 functions as a central regulator of plant defense, ABA-mediated leaf senescence, hormone metabolism and light-mediated seedling development (Delessert et al., 2005; Wang et al., 2009; Huh et al., 2012; Wang and Culver, 2012; Peng et al., 2015). All seven SNAC genes from Arabidopsis are induced by long-term treatment with ABA and/or during age-dependent senescence (Takasaki et al., 2015). *GmNAC030* shares several biological properties with *ATAF1* and *ATAF2*. It is induced by abiotic stress, biotic stress, hormone signaling and it is also involved in stress-induced and developmentally programmed leaf senescence (Mendes et al., 2013; Carvalho et al., 2014b; Pimenta et al., 2016). The SNAC-A member AT5G22380 (ANAC090) is also involved in senescence as part of a senescence-regulating NAC tripartite module (Kim et al., 2018). Likewise, GmNAC011 (Glyma.13G030900, firstly described as GmNAC020) belongs to the SNAC-A subfamily and has been described as a stress-responsive protein, which confers salt and freezing tolerance and is involved in lateral root formation (Hao et al., 2011). Other members of the SNAC-A subfamily, including GmNAC035 (Glyma.06G1140000), GmNAC011 (Glyma.13G030900), GmNAC109 (Glyma.14G152700), and GmNAC039 (Glyma.06G157400), have shown to display a stress-induced expression profile consistent with their clustering into this family (Tran et al., 2009).

Another component of the stress-induced NRP/DCD-mediated signaling, GmNAC081 (Glyma.12G022700; Faria et al., 2011), was clustered in the TERN (Tobacco elicitor-responsive gene encoding NAC-domain protein) subfamily, which encompasses functionally similar NAC proteins that are involved in resistance to biotic and abiotic stresses. Examples from this subfamily include (i) *Gossypium barbadense* (Gb)NAC1, which is involved in the positive regulation of resistance to Verticillium and in abiotic stress response in cotton (Wang et al., 2016), (ii) the chitin-responsive gene *AT3G44350 (ANAC061*) and (iii) *AT2G17040* (*ANAC036)*, an orthologous gene of *GmNAC081* (Libault et al., 2007; Reis et al., 2016). GmNAC081 has been shown to regulate stress-induced and developmentally programmed leaf senescence (Mendes et al., 2013; Pimenta et al., 2016).

GmNAC124 (Glyma.16G043200, firstly described as GmNAC11) belongs to the SNAC-B (NAP). Accordingly, it is induced by stress and confers salt tolerance (Hao et al., 2011). The founding member of the SNAC-B (NAP) subfamily, the NAC-like activated by AP3/PI (AtNAP, AT1G69490), is a crucial regulator of leaf senescence and couples ABA biosynthesis with chlorophyll loss (Yang et al., 2014c). *GmNAC006* (Glyma.02G070000), which also clusters with members of this family, has been shown to be salt- and dehydration-induced (Tran et al., 2009). GmNAC representatives of the vascular-related NAC domain (VND-NAC) subfamily include GmNAC048 (Glyma.07G05060) and GmNAC122 (Glyma.16G019400), functionally characterized as orthologs of the Arabidopsis NAC secondary wall thickening promoting factor 1, NST1 (AT2G46770), NST2 (AT3G61910), and NST3 (AT1G32770) (Mitsuda et al., 2005, 2007; Mitsuda and Ohme-Takagi, 2008; Dong et al., 2013). In Arabidopsis, members of this family are very well characterized NAC proteins, involved in the control of vascular differentiation, including also the vascular-related NAC domain 6 (VND6) and VND7 (Yamaguchi et al., 2010, 2011). These examples of characterized members of the soybean NAC family substantiate the notion that NACs of the same subfamily from different species conserve similar function, expression, and sequence.

### Stress- and Senescence-Associated Expression Analyses Confirm the Phylogenetic Classification of the New GmNAC Genes

We also showed that a fraction of the novel NAC genes responds to at least one of the stimuli tested, including ER stress (TUN treatment), osmotic stress (PEG treatment), SA treatment, or developmentally programmed leaf senescence. Although not functionally characterized, these new, previously unidentified, GmNACs displayed expression profiles consistent with their subfamily classification, which may extend to accommodate similar function. Accordingly, GmNAC154 (Glyma.02G284300), a member of the ONAC022 subfamily, shares a high degree of conservation with ANA042 (JUB1; AT2G4300000), which has been described as a negative regulator of important growth repressors and key genes involved in gibberellin (GA), brassinosteroid (BR) biosynthesis and senescence (Wu et al., 2012; Shahnejat-Bushehri et al., 2016). Besides its developmental function, JUB1 displays other functions in promoting tolerance to drought, heat and salinity (Wu et al., 2012; Shahnejat-Bushehri et al., 2016; Ebrahimian-Motlagh et al., 2017). *JUB1* is up-regulated by H_2_O_2_ -mediated oxidative stress, which may explain its dual involvement with development and stress response. In fact, JUB1 has been implicated as a central regulator of a finely tuned control system that modulates the cellular H_2_O_2_ level and primes the plants for upcoming stress through a gene regulatory network that involves DREB2A (Wu et al., 2012). Accordingly, the tomato *JUB1* ortholog has also been shown to be induced by H_2_O_2_, NaCl, osmotic stress and leaf dehydration and to enhance drought tolerance in transgenic tomato lines (Thirumalaikumar et al., 2017). In addition to sharing similar expression profiles, the abiotic stress-related gene regulatory networks controlled by tomato and Arabidopsis JUB1 have been demonstrated to be highly conserved. The expression profile of GmNAC154 indicated that this soybean NAC might be a JUB1 ortholog as it is controlled by biotic and abiotic stresses, which are triggered by H_2_O_2_ accumulation as the common denominator.

*GmNAC163* (Glyma.06G288500), which was shown to be strongly up-regulated by PEG, TUN and SA, belongs to the ANAC001 subfamily and is closely related to ANAC073/SND2 (AT4G28500) that regulates genes involved in secondary cell wall development. ANAC073 controls the expression of cellulose and hemicellulose biosynthetic genes in addition to those involved in lignin polymerization and signaling (Hussey et al., 2011).

*GmNAC174* (Glyma.12G186900) and *GmNAC177* (Glyma.16G0164000), which belong to different phylogenetic subfamilies, were rapidly induced by TUN and SA. GmNAC174 (subfamily OsNAC8) is closely related to ANAC089 (AT5G22290) a membrane-tethered transcription factor that negatively regulates floral initiation. ANAC089 also controls ER-stress-induced programmed cell death and is induced by TUN, like the predicted transmembrane soybean ortholog GmNAC174 (Yang et al., 2014b). Under severe ER stress conditions, the membrane-associated transcription factor ANAC089 relocates from the ER membrane to the nucleus to induce programmed cell death. *GmNAC177*, also induced by TUN and SA, belongs to an unnamed monophyletic group of soybean NAC genes, that cluster very closely to the TIP subfamily. Proteins from the TIP subfamily are involved in senescence progression and immune response control (Pinheiro et al., 2009; Wang et al., 2009; Block et al., 2014). Specifically, AT4G35580 has been demonstrated to interact with the *Pseudomonas syringae* type III effector HopD1 in the endoplasmic reticulum to suppress effector-triggered immunity (Block et al., 2014). Hence, it is not surprising that *GmNAC177*, a soybean close relative of AT4G35580, is regulated by ER stress and SA. As a representative of the novel GmNACs, GmNAC177 was further characterized and showed that displays transcriptional activity in yeast, is nuclear localized and induces cell death *in planta*, which is consistent with its close relatedness with the TIP subfamily.

The *GmNAC065* (Glyma.08G307100) orthologous gene from Arabidopsis, *ANAC083* (*AT5G13180*), also designated VND-interacting 2 (*VNI2*), interacts with VND7 and negatively regulates xylem vessel formation in Arabidopsis (Yamaguchi et al., 2010). In addition, VNI2 has been shown to integrate ABA-mediated abiotic stress signals into leaf aging by regulating a subset of COLD-REGULATED (COR) and RESPONSIVE TO DEHYDRATION (RD) genes (Yang et al., 2011). The *VNI2* gene is induced by high salinity in an ABA-dependent manner and displays spatial and temporal expression patterns correlated with leaf aging and senescence. VNI2 may also be involved in biotic stress responses as it has been shown to interact with the geminiviral replication initiator protein (Rep) from *Mungbean yellow mosaic India virus* (Suyal et al., 2014). We showed here that *GmNAC065* share developmental and stress response similarly to its orthologous gene VNI2 and may play a similar function related to cell death events.

*GmNAC085* (Glyma.12G149100) belongs to the SNAC-A (ATAF) subfamily, which contains seven homologous genes from Arabidopsis. All seven SNAC-A genes from Arabidopsis, *ANAC055* (AT3G15500), *ANAC019* (AT1G52890), *ANAC072/RD26* (AT4G27410), *ANAC002/ATAF1* (AT1G01720), *ANAC081/ATAF2* (AT5G08790), *ANAC102* (AT5G63790), and *ANAC032* (AT1G77450), are induced during age-dependent senescence; thereby, up-regulating ABA- and senescence-inducible genes and play crucial roles in ABA-induced leaf senescence signaling (Takasaki et al., 2015). Likewise, 90% of the orthologous SNAC-A (ATAF) genes from soybean were differentially expressed during natural leaf senescence (Supplementary Table 6). The most closely related protein of GmNAC085, ANAC072 (AT4G27410), also designated RD26, RESPONSIVE TO DESICCATION 26, acts as a transcriptional activator in ABA-mediated dehydration response. ANAC072 positively regulates both age- and dark-induced leaf senescence through activating the transcription of NYE1, a key regulatory gene in chlorophyll degradation (Li et al., 2016a). Except for PEG treatment, *GmNAC085* did not respond to TUN and SA treatments, and in contrast to the SNAC-A genes from Arabidopsis, which are induced during leaf senescence, *GmNAC085* was repressed by developmentally programmed leaf senescence. Among the SNAC-A proteins, GmNAC085 forms a more divergent and separate clade along with other three GmNACs, which may explain a different expression profile and functional divergence from characterizing SNAC-As.

In summary, our results represent an update of the NAC inventory in the soybean genome. Furthermore, our results demonstrated that a representative sample of the novel, previously unidentified transcription factors of the GmNAC superfamily is expressed in response to stress conditions or a senescence signal, and their regulation may be necessary for elicitation of diverse response pathways triggered by different stimuli. The expression profiles of newly identified NAC genes and functional characterization of previously uncharacterized ones validate the genome annotation and reinforce the plasticity of biological functions played by GmNACs. The soybean GmNACs were separated into 15 subfamilies based on phylogeny relatedness, showing a clear relationship between structure, expression, and function for representatives of each subfamily. The large number of proteins encoded by this family and their great diversity allow plants to elaborate complex responses, with gradual levels of hormonal, temporal and spatial regulation with the high plasticity of responses, which, nevertheless, may be anticipated with a high degree of ascertaining by phylogenetic relatedness of the GmNAC proteins.

## Author Contributions

BM conducted all experiments, analyzed data and wrote the first draft of the manuscript. OF performed qRT-PCR. JS and OB performed the bioinformatics and RNA-seq analyses. DF, PC, and JM carried out RNA-seq. PR designed experiments. EF designed experiments, analyzed data and wrote the manuscript. All authors contributed to the discussion and approved the final manuscript.

### Conflict of Interest Statement

The authors declare that the research was conducted in the absence of any commercial or financial relationships that could be construed as a potential conflict of interest.
