# Supplementary material for: Revisiting the Soybean GmNAC Superfamily
Source: Front Plant Sci. 2018 Dec 18;9:1864. doi: 10.3389/fpls.2018.01864 (PMC6305603; doi:10.3389/fpls.2018.01864)
Supplement: Supplementary file 1 [file Data_Sheet_1.PDF]

Supplementary Figures

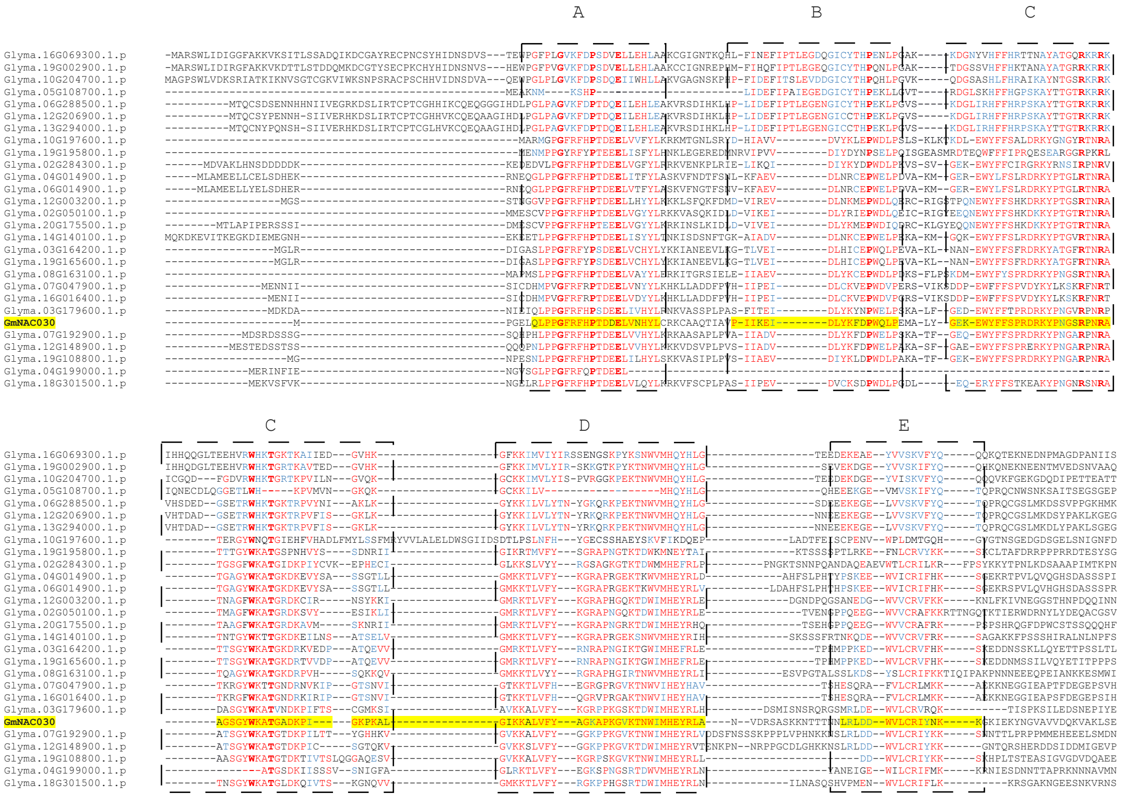

Supplementary Figure 1. Structural organization of the NAC domain of newly identified GmNACs. The multiple sequence alignment of the N-terminal of 27 new GmNACs shows the NAC domain, which is sub-divided into the five motifs (A to E) delimited by the dashboards. The most frequent amino acids are shown in red and the second most frequents in blue. Conserved amino acids are shown in bold. GmNAC030 (yellow) was used as a guide for the multiple sequence alignment.

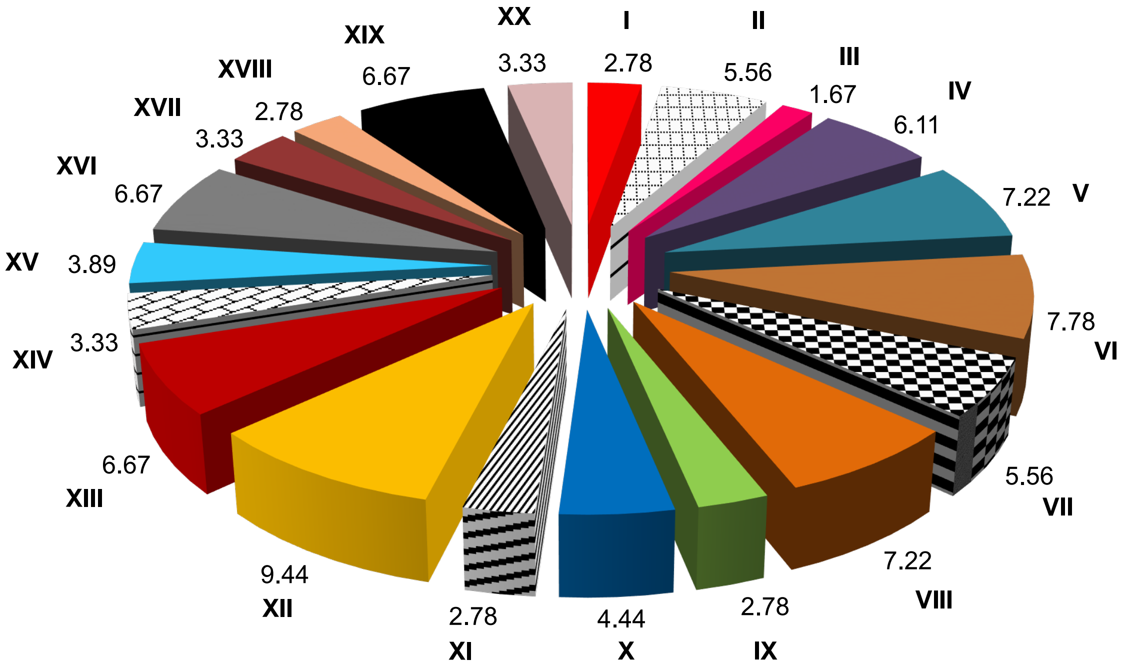

Supplementary Figure 2. Percentage of NAC genes located in each soybean chromosome. The sectorial graphic presents the proportion (%) of the distribution of NAC genes in all 20 soybean chromosomes.

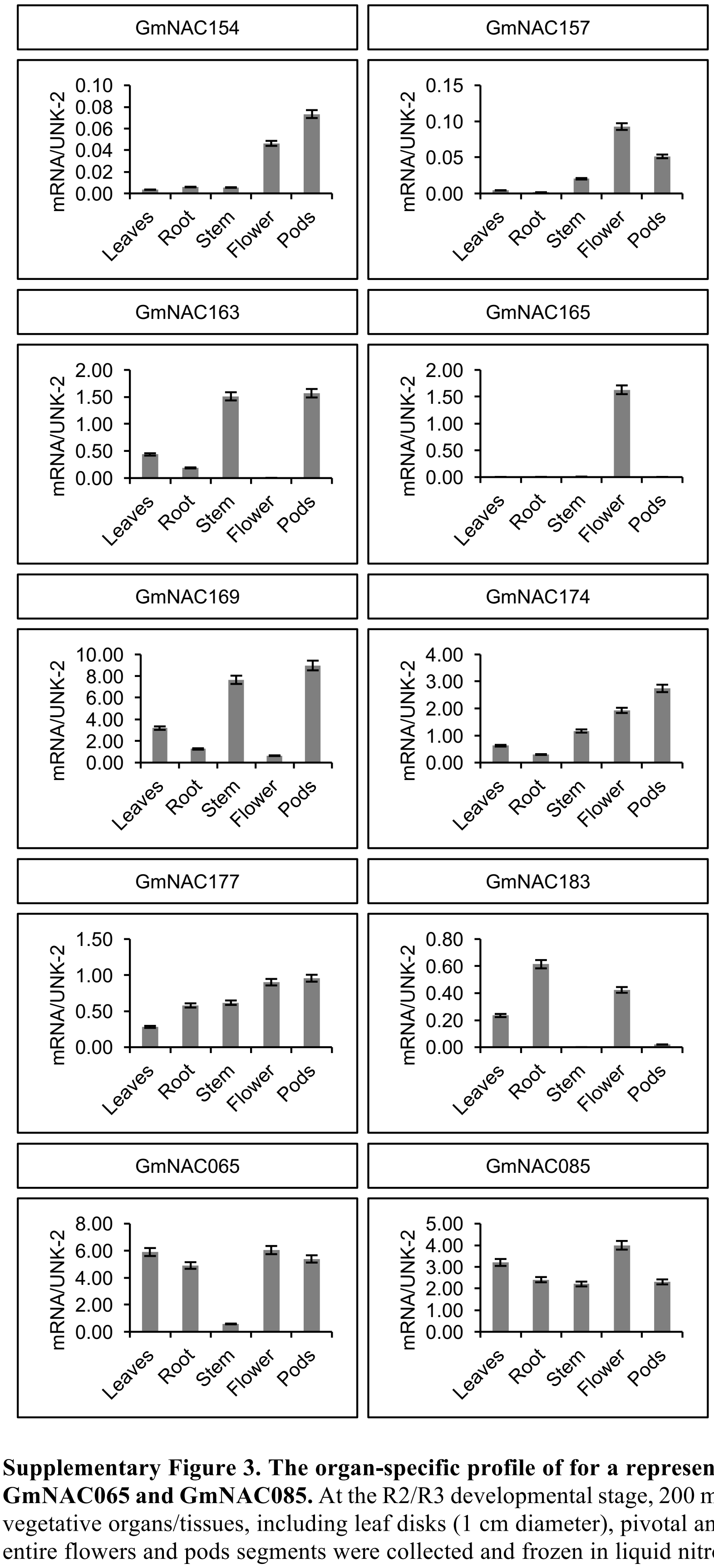

Supplementary Figure 3. The organ-specific profile of for a representative sample of the new NAC genes, GmNAC065 and GmNAC085. At the R2/R3 developmental stage, 200 mg of fresh tissue from different soybean vegetative organs/tissues, including leaf disks (1 cm diameter), pivotal and lateral roots, stem's segments (1 cm), entire flowers and pods segments were collected and frozen in liquid nitrogen. Total RNA was isolated from the indicated organs, and the transcript accumulation of the indicated genes was measured by qRT-PCR. U*KN-2* was chosen as the normalizer, endogenous control gene. Gene expression was quantified using the e $2^{-\Delta Ct}$ method. Data were obtained from the average of 3 biological samples (pools of 5 plants) and 2 technical replicates for each treatment. The bars indicate standard error.

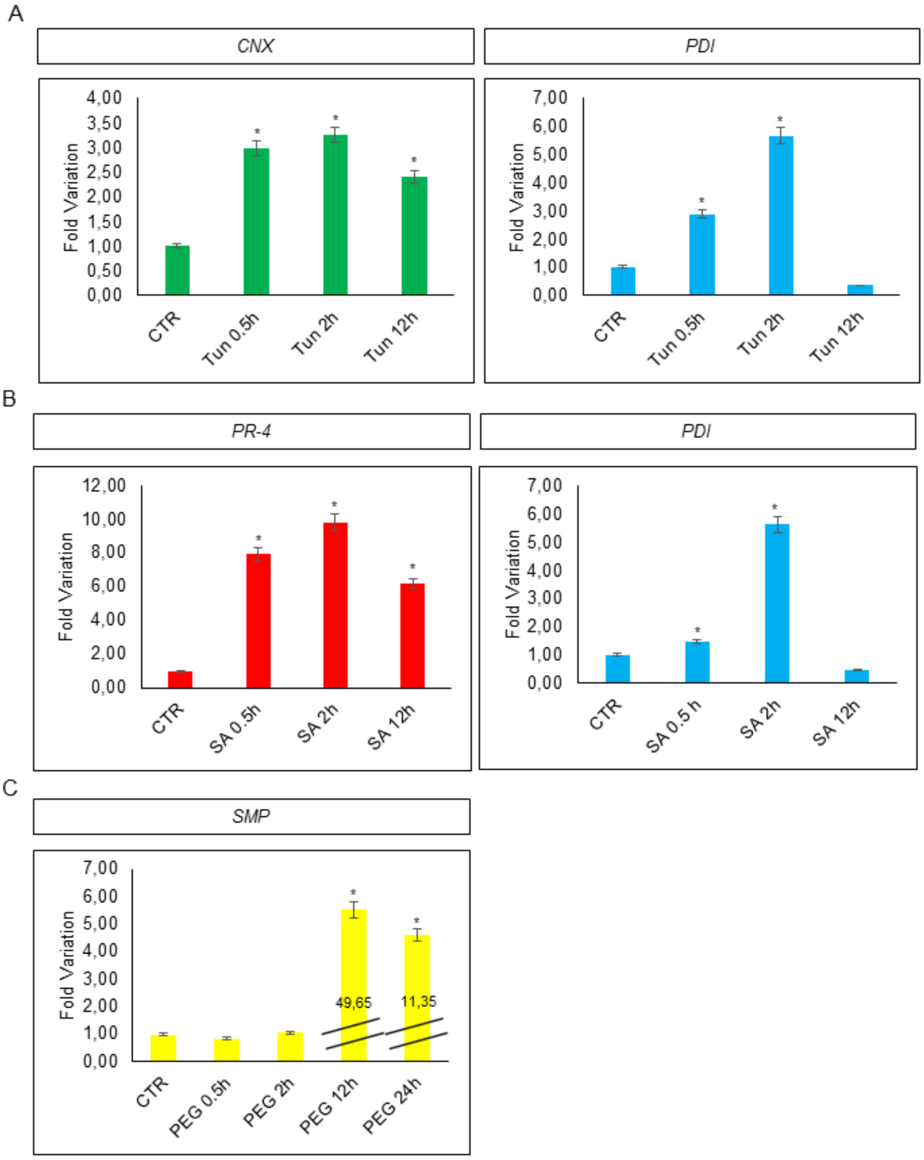

Supplementary Figure 4. Expression of stress-associated marker genes in soybean seedlings. (A) Calnexin (*CNX*) and Protein–dissulfide isomerase (*PDI)* gene expression during tunicamicyn treatment. **(B)** Pathogenesis-related gene 4 (*PR-4*) and *PDI* gene expression during SA treatment. **(C)** Seed maturation protein (*SMP*) expression during PEG treatment. Dimethyl sulfoxide (DMSO) was used as a control for tunicamycin treatment. Leaf disks from stressed and control leaves were collected at 0.5, 2 and 12 h post-treatment (for PEG treatment, 24-h harvest time was included). *UKN-2* was chosen as the normalizer, endogenous control gene. Relative gene expression was quantified using the comparative $2^{-\Delta\Delta Ct}$ method. The bars indicate standard-error and the asterisks indicate statistical significance by the t-test, ($P < 0.05$, $n = 3$).

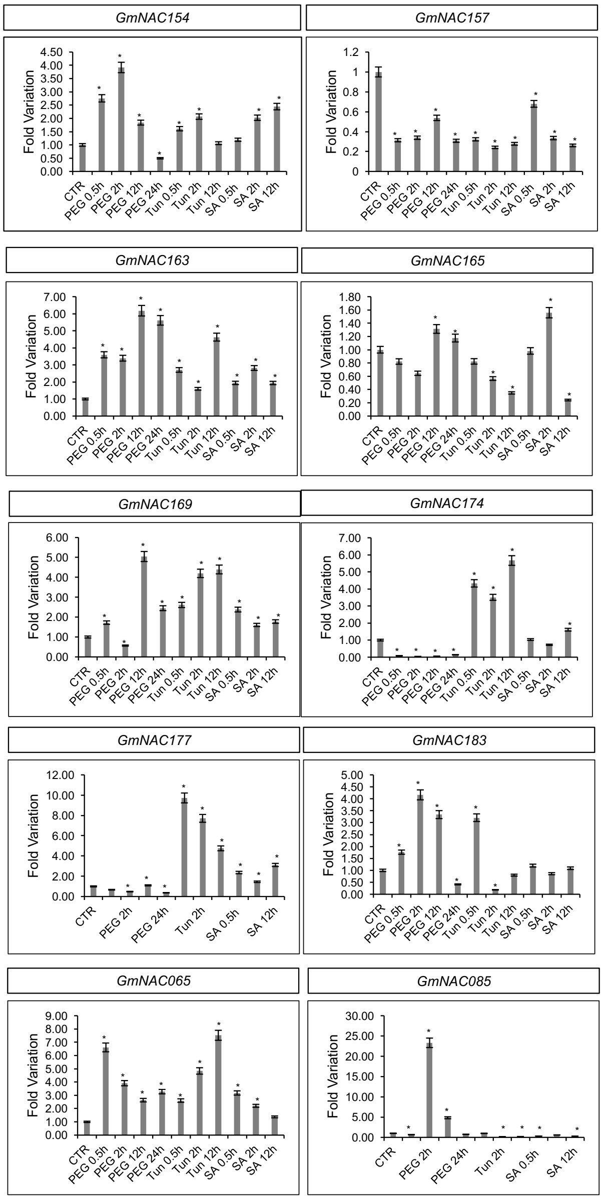

Supplementary Figure 5. The stress-induced expression profile for a representative sample of the new NAC genes, *GmNAC065 and GmNAC085*. Expression profile of 8 new putative NAC genes: GmNAC154 (ONAC022), GmNAC157 (NAM), GmNAC163 (ANAC001), GmNAC165 (SNAC-B - NAP), GmNAC169 (NAM), GmNAC174 (OsNAC8), GmNAC177 (unnamed), GmNAC 183 (TERN), GmNAC065 (Senu5) and GmNAC085 (SNAC-A – ATAF) during multiple stress. At the V2/V3 developmental stage, the roots were immersed in Hoagland Hydroponic solution supplemented with 10% (w/v) PEG (MW 8,000), 5 µg/mL tunicamycin or 5 mM salicylic acid (SA) to induce osmotic, endoplasmic reticulum and biotic stress conditions, respectively. Dimethyl sulfoxide (DMSO) was used as a control for tunicamycin treatment. Leaf disks from stressed and control leaves were collected at 0.5, 2 and 12 h post-treatment (for PEG treatment, 24-h harvest time was included). *UKN-2* was chosen as the normalizer, endogenous control gene. Relative gene expression was quantified using the comparative $2^{-\Delta\Delta Ct}$ method. The bars indicate standard-error and the asterisks indicate statistical significance by the t-test, ($P < 0.05$, $n = 3$).

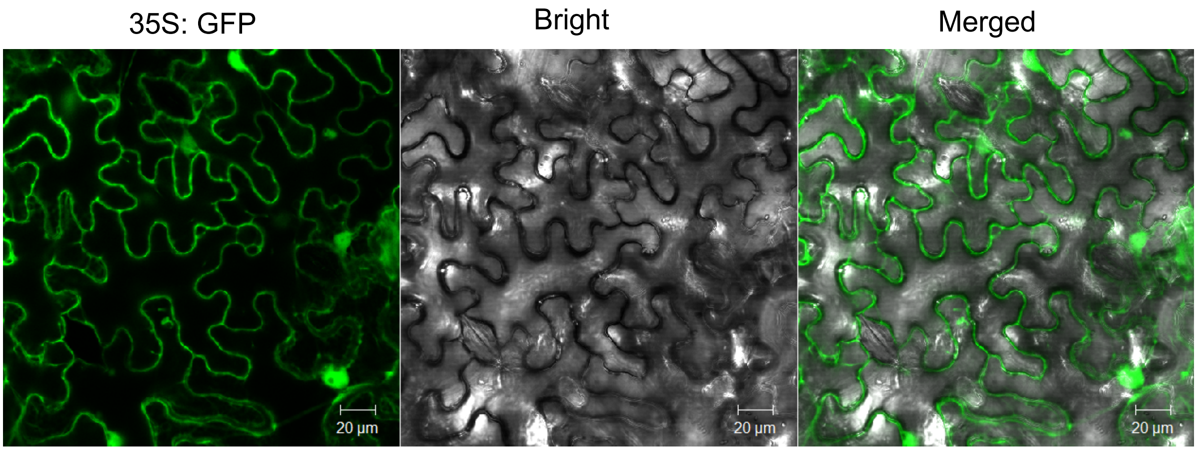

Supplementary Figure 6. Confocal fluorescence image of transiently expressed GFP in epidermal cells of *N. benthamiana* leaves. The conditions of GFP expression and imaging was as described in Figure 8. Scale bars, 20 mm.
