# Supplementary material for: Revisiting the Soybean GmNAC Superfamily
Source: Front Plant Sci. 2018 Dec 18;9:1864. doi: 10.3389/fpls.2018.01864 (PMC6305603; doi:10.3389/fpls.2018.01864)
Supplement: Supplementary file 3 [file Data_Sheet_3.docx]

**Table S2. PCR-primers used for cloning the NAC genes**

| **Primers** | **Sequence (5’ - 3’)** | **Gene identity** |
| --- | --- | --- |
| GlymaNAC85 fwd | AAAAAGCAGGCTTCACAATGGGAAGTTCAGAGAGAGA | Glyma.12G149100 |
| GlymaNAC85 rvs | AGAAAGCTGGGTCTCAGTCCCCTAAACCCGAACTC | Glyma.12G149100 |
| GlymaNAC65 fwd | AAAAAGCAGGCTTCACAATGGAGAAGGTGAAATTTTGTG | Glyma.08G360200 |
| GlymaNAC65 rvs | AGAAAGCTGGGTCTTCTAATAGTATAAGGGAAGG | Glyma.08G360200 |
| NAC177fwd | AAAAGCAGGCTTCACAATGGAGAACATAATTTCCATATGTGGATC | Glyma.16G016400 |
| NAC177rvs | AGAAAGCTGGGTCGGGTATGAACTCATCTTCGTCATCCGTAACT | Glyma.16G016400 |
| attB1 | GGGGACAAGTTTG TACAAAAAAGCAGGCT | - |
| attB2 | GGGGACCACTTTGTA CAAGAAAGCTGGGT | - |

**Table S3. Recombinant plasmids.**

| **Clone Name** | **Description*** |
| --- | --- |
| pUFV2589 | GmNAC065 ORF was isolated from 48h-tunicamycin-stressed leaf cDNA and cloned into pDONR201. |
| pUFV2770 | GmNAC065 was transferred from pUFV2589 to pDEST32 through LR clonase recombination. In the resulting clone,GmNAC065 was fused to the GAL4 binding domain (BD-NAC065). |
| pUFV2780 | GmNAC065 was transferred from UFV2589 to pDEST22 through recombination. In the resulting clone GmNAC065 was fused to the GAL4 activating domain (AD-NAC0065) |
| pUFV2826 | GmNAC177 ORF was isolated from a pool of cDNA from tunicamycin-, PEG- and salicylic acid-stressed leaves and cloned into pDONR201. |
| pUFV2827 | GmNAC085 ORF was isolated from a pool of cDNA from tunicamycin-, PEG- and salicylic acid-stressed leaves and cloned into pDONR207. |
| pUFV2828 | GmNAC177 was transferred from pUFV2826 to pDEST32 by recombination (BD-NAC117) |
| pUFV2829 | GmNAC085 was transferred from pUFV2827 to pDEST32 through LR clonase recombination reaction using the clone 2827 (BD-NAC85). |
| pUFV2830 | GmNAC177was transferred from pUFV2826 to pEARLEY103 through LR clonase recombination. The resulting clone expresses NAC177-GFP fusion, under the control of 35S promoter. |
| pUFV3007 | GmNAC065 was transferred from pUFV3013 to pEARLEY103 through recombination. The resulting clone expresses NAC065-GFP protein fusion, under the control of 35S promoter. |
| pUFV3008 | GmNAC085 was transferred from pUFV2827 to pERALEY104 by recombination. The resulting clone expresses YFP-NAC85 protein fusion, under the control of the 35S promoter. |
| pUFV3009 | GmNAC085 ORF was amplified from pUFV2827 and cloned into pDONR201 by recombination. |
| pUFV3010 | GmNAC085 ORF was transferred from pUFV3009 to pDEST22 by recombination, generating AD-NAC085 fusion. |
| pUFV3011 | GmNAC177 ORF was amplified from pUFV2826 and cloned into pDONR 207 by recombination. |
| pUFV3012 | GmNAC177 ORF was transferred from pUFV2826 to pDEST22 through recombination, generating AD-NAC117 fusion. |
| pUFV3013 | GmNAC065 ORF was amplified from pUFV2589 and cloned into pDONR 207 by recombination. |

*All recombinant plasmids were obtained through the GATEWAY system.

**TabeS4.Primers for qRT-PCR.**

| **Primer** | **Sequence (5’ - 3’)** | **Gene Identity** |
| --- | --- | --- |
| CNXfwd | TGATGGGGAGGAGAAGAAAAAGGC | Glyma.05G199200 |
| CNXrvs | CGGTGTAGACATGGGAAAGC | Glyma.05G199200 |
| PDIfwd | TTGGTTGAAGGCGGTACAAGGATGG | Glyma.10G217600 |
| PDIrvs | ACTCCAGCAGAACTATCTTCCCAG | Glyma.10G217600 |
| PR-4fwd | TGCGGGTGACAAATACAGGA | Glyma.19G245400 |
| PR-4rvs | TGCTGCACTGATCTACGATTCTC | Glyma.19G245400 |
| qRTNAC177fwd | AAATCCGGCAAGAGCAGAAG | Glyma.16G016400 |
| qRTNAC177rvs | CCACTGCCCGAAGATTTCA | Glyma.16G016400 |
| qRTNAC154fwd | GTAGCTCAGGCTCCAACATCC | Glyma.02G284300 |
| qRTNAC154rvs | ACCACAGACGTGAGATCATCC | Glyma.02G284300 |
| qRTNAC157fwd | GGACATTCAGATGCTTCTTCGTC | Glyma.04G014900 |
| qRTNAC157rvs | AAAGATGAGGGTGAG AGAGGC | Glyma.04G014900 |
| qRTNAC163fwd | GGTCAAAGTGTGCATGTTGAGG | Glyma.06G288500 |
| qRTNAC163rvs | TTGAGCATTTTGCCCTCCTT | Glyma.06G288500 |
| qRTNAC165fwd | TCC CTG CTA AGC CAG TTT CC | Glyma.07G192900 |
| qRTNAC165rvs | ATG AAA TTG TTG CCT CGG CG | Glyma.07G192900 |
| qRTNAC169fwd | CTGGATGCCAACGAGAATC | Glyma.10G197600 |
| qRTNAC169rvs | TATCCCCATTCCCATTGCA | Glyma.10G197600 |
| qRTNAC174fwd | GGGAGAGGACCGAATGGATTA | Glyma.12G186900 |
| qRTNAC174rvs | CTTGAGGCGACAAATGACCAA | Glyma.12G186900 |
| qRTNAC183fwd | AGC AGA GAG CTC TTC CTC CG | Glyma.19G195800 |
| qRTNAC183rvs | CCA AGA ACA TAT CCA CTT TCT CC | Glyma.19G195800 |
| qRTNAC65fwd | TGGGATTTGCCAGGTGATTT | Glyma.08G360200 |
| qRTNAC65rvs | GAGCGATTTCCGTTGGGATA | Glyma.08G360200 |
| qRTNAC85fwd | CAGCAGCAGGACGAGAAATTC | Glyma.12G149100 |
| qRTNAC85rvs | TCAAGATCCGTCGGGTTGAC | Glyma.12G149100 |
| SMPfwd | GCCGAACTGAGGAAAAGACGAACC | Glyma.20G147500 |
| SMPrvs | CTTGGGCTGTTTGTTGGTCTTC | Glyma.20G147500 |
| UNK-2fwd | GCCTCTGGATACCTGCTCAAG | Glyma.06G041800 |
| UNK-2rvs | ACCTCCTCCTCAAACTCCTCTG | Glyma.06G041800 |

* The primers were designed by PrimerExpress 3.0 software to minimize performance-penalties
